# Adapting (4,4) Networks through Substituent Effects and Conformationally Flexible 3,2’:6’,3”-Terpyridines

**DOI:** 10.3390/molecules26216337

**Published:** 2021-10-20

**Authors:** Dalila Rocco, Alessandro Prescimone, Edwin C. Constable, Catherine E. Housecroft

**Affiliations:** Department of Chemistry, University of Basel, BPR 1096, Mattenstrasse 24a, CH-4058 Basel, Switzerland; dalila.rocco@unibas.ch (D.R.); alessandro.prescimone@unibas.ch (A.P.); edwin.constable@unibas.ch (E.C.C.)

**Keywords:** cobalt, 3,2’:6’,3”-terpyridine, [1,1’-biphenyl]-4-yl substituents, 2-dimensional network, coordination polymer, crystal structure

## Abstract

Coordination networks formed between Co(NCS)_2_ and 4’-substituted-[1,1’-biphenyl]-4-yl-3,2’:6’,3”-terpyridines in which the 4’-group is Me (**1**), H (**2**), F (**3**), Cl (**4**) or Br (**5**) are reported. [Co(**1**)_2_(NCS)_2_]*_n_*·4.5*n*CHCl_3_, [Co(**2**)_2_(NCS)_2_]*_n_*·4.3*n*CHCl_3_, [Co(**3**)_2_(NCS)_2_]*_n_*·4*n*CHCl_3_, [Co(**4**)_2_(NCS)_2_]*_n_*, and [Co(**5**)_2_(NCS)_2_]*_n_*·*n*CHCl_3_ are 2D-networks directed by 4-connecting cobalt nodes. Changes in the conformation of the 3,2’:6’,3”-tpy unit coupled with the different peripheral substituents lead to three structure types. In [Co(**1**)_2_(NCS)_2_]*_n_*·4.5*n*CHCl_3_, [Co(**2**)_2_(NCS)_2_]*_n_*·4.3*n*CHCl_3_, [Co(**3**)_2_(NCS)_2_]*_n_*·4*n*CHCl_3_, cone-like arrangements of [1,1’-biphenyl]-4-yl units pack through pyridine…arene π-stacking, whereas Cl…π interactions are dominant in the packing in [Co(**4**)_2_(NCS)_2_]*_n_.* The introduction of the Br substituent in ligand **5** switches off both face-to-face π-stacking and halogen…π-interactions, and the packing interactions are more subtly controlled. Assemblies with organic linkers **1**–**3** are structurally similar and the lattice accommodates CHCl_3_ molecules in distinct cavities; thermogravimetric analysis confirmed that half the solvent in [Co(**3**)_2_(NCS)_2_]*_n_*·4*n*CHCl_3_ can be reversibly removed.

## 1. Introduction

**Scheme 1:**
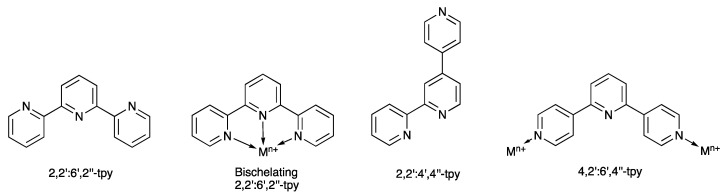
Conformations of free 2,2’:6’,2”-tpy and of the terdentate chelating ligand. Structure of 4,2’:6’,4”-tpy, an example of another terpyridine isomer, which retains a bpy domain.

**Scheme 2:**
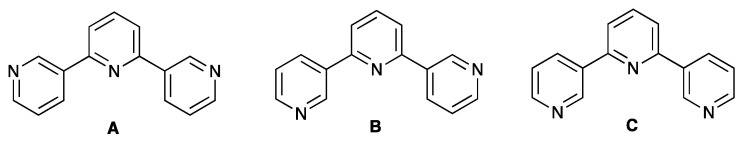
Limiting planar conformations of 3,2’:6’,3”-terpyridine.

**Scheme 3:**
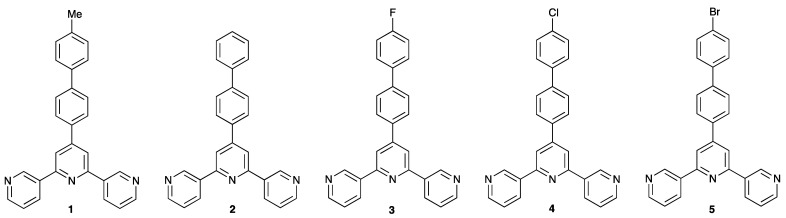
Structures of compounds **1**–**5** with the 3,2’:6’,3’’-tpy unit shown in one of the three possible planar conformations (see Scheme 2).

The coordination chemistry of terpyridine (tpy) ligands is dominated by that of the chelating 2,2’:6’,2”-tpy, which undergoes a conformational change from *s-trans,s-trans* to *s-cis*,*s-cis* upon binding a metal ion (Scheme 1) [1,2,3,4,5]. A further 47 isomers of tpy can be drawn, and eleven of these retain a chelating 2,2’-bipyridine (bpy) metal-binding domain (e.g., 2,2’:4’,4”-tpy, Scheme 1) [6]. 4,2’:6’,4”-Terpyridine (4,2’:6’,4”-tpy) and 3,2’:6’,3”-terpyridine (3,2’:6’,3”-tpy) are noteworthy in being divergent building blocks, appropriate for the formation of coordination polymers and networks [7,8]. Interestingly, these ligands only coordinate to metal ions through the outer pyridine rings. While 4,2’:6’,4”-tpy presents a V-shaped linker between metal centres (Scheme 1), 3,2’:6’,3”-tpy is conformationally flexible by virtue of inter-ring C–C bond rotation [7,9,10,11,12]. The three limiting planar conformations of 3,2’:6’,3”-tpy are shown in Scheme 2. The vectorial properties of the lone pairs of the outer N atoms in conformations **A** and **B** are divergent and, therefore, suited to the assembly of coordination polymers [7]. In contrast, conformation **C** is often observed in discrete molecular structures [13,14,15,16,17].

The synthetic ease of introducing functional groups into the 4’-position of symmetrical terpyridines (2,2’:6’,2”-tpy, 3,2’:6’,3”-tpy and 4,2’:6’,4”-tpy) by using either a Kröhnke synthesis [18] or the one-pot strategy of Wang and Hanan [19] is attractive in terms of undertaking systematic investigations of the steric and electronic effects of functional groups on the solid-state structures of coordination compounds. For example, combinations of zinc(II) acetate and 4’-(4-*n*-alkyloxyphenyl)-4,2’:6’,4”-terpyridines in which the alkyl group, R, is a member of the homologous series from methyl to *^n^*decyl lead to 1D-coordination polymers for small substituents. In contrast, discrete [Zn_2_(μ-OAc)_4_(4′-(4-ROC_6_H_4_)-4,2’:6’,4”-tpy)_2_] complexes are formed for the longest alkyl chains, and competition between molecular and infinite assemblies occurs for intermediate *n*-alkyloxy groups [20]. We have also investigated the effects of the length of the alkyl chain in [CoL_2_(NCS)_2_]*_n_* 2D-networks in which L is a 4’-(4-*n*-alkyloxyphenyl)-3,2’:6’,3”-terpyridine, and have demonstrated that an increase in the *n*-alkyloxy chain length results in a change in the conformation of the 3,2’:6’,3”-tpy metal-binding domain [10]. This investigation was extended to isomeric C_4_-alkyloxy substituents, and revealed significant structural perturbation on going from *n*-butyl to branched isomers, but only subtle changes across the series *rac*-4-butan-2-yl, 2-methylpropyl and *tert-*butyl peripheral groups [21]. We have also investigated the effects of introducing different halogen-substituents in 3,2’:6’,3”-tpy and 4,2’:6’,4”-tpy ligands containing 4’-biphenyl-4-yl substituents [12,22]. The most recent of these studies focused on ligands **1**–**5** (Scheme 3) and revealed that the 1D-coordination polymers [Cu_2_(μ-OAc)_4_(**2**)]*_n_* and [Cu_2_(μ-OAc)_4_(**3**)]*_n_* are isostructural featuring inter-chain H…H and H…F interactions, respectively. In contrast, the greater steric demands of the Me, Cl or Br substituents in [Cu_2_(μ-OAc)_4_(**1**)]*_n_*, [Cu_2_(μ-OAc)_4_(**4**)]*_n_* and [Cu_2_(μ-OAc)_4_(**5**)]*_n_* resulted in a change in the conformation of the 3,2’:6’,3”-tpy unit from **A** to **B** (Scheme 2) coupled with a change in inter-chain π-stacking interactions [12]. We now report how the structures of the 2D-networks formed in reactions of Co(NCS)_2_ with ligands **1**–**5** are influenced by the peripheral substituent (Me, H, F, Cl or Br).

## 2. Results and Discussion

### 2.1. Crystal Growth and Bulk Material Characterization

Ligands **1**–**5** were prepared as previously reported [12]. Reactions between Co(NCS)_2_ and each ligand were carried out under ambient conditions by layering a methanol solution of Co(NCS)_2_ over a chloroform solution of **1**, **2**, **3**, **4** or **5**. Depending on the ligand, single crystals of X-ray quality were obtained within one day and two weeks (see Section 3.2, Section 3.3, Section 3.4, Section 3.5 and Section 3.6), and structures were determined for [Co(**1**)_2_(NCS)_2_]*_n_*·4.5*n*CHCl_3_, [Co(**2**)_2_(NCS)_2_]*_n_*·4.3*n*CHCl_3_, [Co(**3**)_2_(NCS)_2_]*_n_*·4*n*CHCl_3_, [Co(**4**)_2_(NCS)_2_]*_n_*, and [Co(**5**)_2_(NCS)_2_]*_n_*·*n*CHCl_3_. All the complexes assemble into 2D-networks with the Co atoms acting as 4-connecting nodes, and the nets may be categorized into three structure types as detailed below.

**Figure 1:**
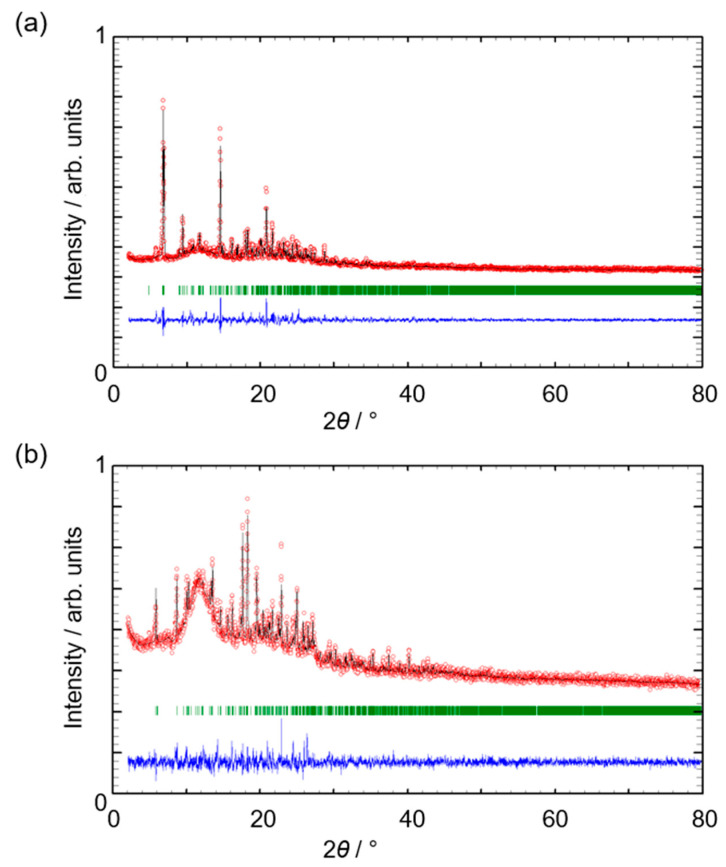
PXRD (CuKα1 radiation) patterns (red circles) of the bulk crystalline materials of (**a**) [Co(**3**)_2_(NCS)_2_]*_n_*·4*n*CHCl_3_, and (**b**) [Co(**5**)_2_(NCS)_2_]*_n_*·*n*CHCl_3_, with fitting to the predicted patterns from the single-crystal structures. The black lines are the best fits from the Rietveld refinements, and green lines show the Bragg peak positions. Each blue plot gives the difference between calculated and experimental points, and the differences in intensities are due to differences in the preferred orientations of the crystallites in the bulk samples.

After selection of a crystal for single crystal X-ray diffraction, the remaining crystals were analyzed by solid-state IR spectroscopy and powder X-ray diffraction (PXRD). The IR spectra are shown in Figures S1–S5 in the Supporting Information. The strongest absorption band in each spectrum is assigned to the thiocyanate C≡N stretching mode (2072 cm^−1^ for [Co(**2**)_2_(NCS)_2_]*_n_*·4.3*n*CHCl_3_, 2070 cm^−1^ for [Co(**1**)_2_(NCS)_2_]*_n_*·4.5*n*CHCl_3_, 2069 cm^−1^ for [Co(**3**)_2_(NCS)_2_]*_n_*·4*n*CHCl_3_ and [Co(**5**)_2_(NCS)_2_]*_n_*·*n*CHCl_3_, and 2063 cm^−1^ for [Co(**4**)_2_(NCS)_2_]*_n_*. Fits between the powder pattern predicted from the single crystal structure and the experimental pattern for [Co(**3**)_2_(NCS)_2_]*_n_*·4*n*CHCl_3_ and [Co(**5**)_2_(NCS)_2_]*_n_*·*n*CHCl_3_ (Figure 1) confirmed that the single crystals were representative of the bulk materials. For the remaining compounds, comparisons of the experimental PXRD and those predicted from the single crystal measurements are shown in Figures S6–S8. Although the correspondence between the PXRD patterns for [Co(**2**)_2_(NCS)_2_]*_n_*·4.3*n*CHCl_3_ (Figure S6) is good, it was not possible to generate a fit using the program FULLPROF (see Section 3.7).

### 2.2. Crystal Structures of [Co(**1**)_2_(NCS)_2_]_n_·4.5nCHCl_3_, [Co(**2**)_2_(NCS)_2_]_n_·4.3nCHCl_3_ and [Co(**3**)_2_(NCS)_2_]_n_·4nCHCl_3_

**Figure 2:**
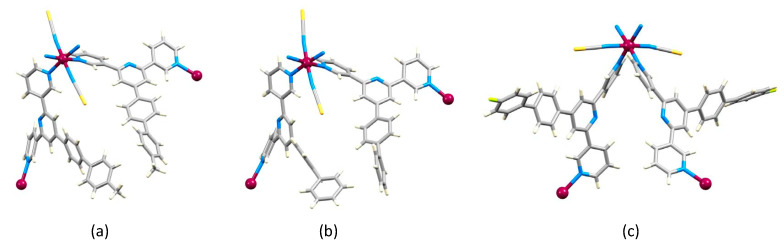
Structures of the asymmetric units in (**a**) [Co(**1**)_2_(NCS)_2_]*_n_*·4.5*n*CHCl_3_, (**b**) [Co(**2**)_2_(NCS)_2_]*_n_*·4.3*n*CHCl_3_, and (**c**) [Co(**3**)_2_(NCS)_2_]*_n_*·4*n*CHCl_3_ with symmetry related atoms. [Co(**3**)_2_(NCS)_2_]*_n_*·4*n*CHCl_3_ contains two independent ligands. Solvent molecules are omitted. For atom numbering, see Figures S9–S11. Co: maroon; N: blue; S: yellow; F: pale green.

**Figure 3:**
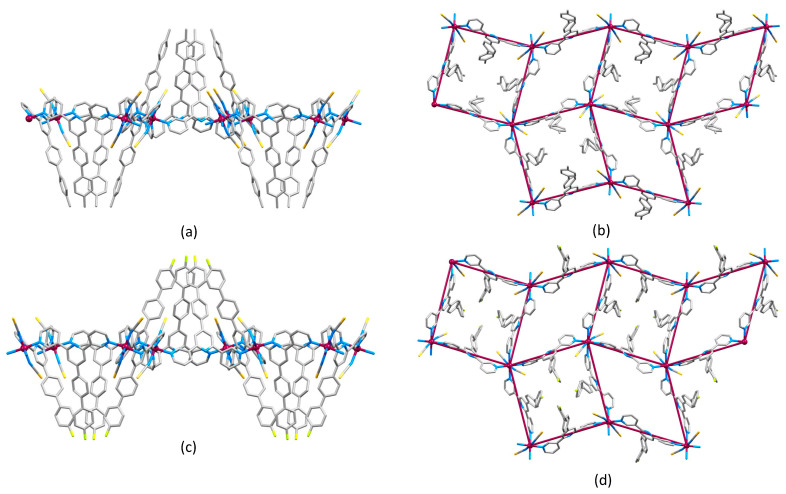
(**a**) Part of one 2D-sheet in [Co(**1**)_2_(NCS)_2_]*_n_*·4.5*n*CHCl_3_ showing cones above and below the plane containing the Co atoms, and (**b**) looking down on the sheet; the cones protrude from the square rhombi. (**c**,**d**) Part of one 2D-sheet in [Co(**3**)_2_(NCS)_2_]*_n_*·4*n*CHCl_3_ showing the cones which project above and below the plane and are built up around the rhombi with internal angles of 87.6° and 92.4°.

[Co(**1**)_2_(NCS)_2_]*_n_*·4.5*n*CHCl_3_ and [Co(**2**)_2_(NCS)_2_]*_n_*·4.3*n*CHCl_3_ crystallize in the tetragonal space group *P*4*/ncc*, while [Co(**3**)_2_(NCS)_2_]*_n_*·4*n*CHCl_3_ crystallizes in the orthorhombic space group *Pccn*. The structures of the asymmetric units of these three compounds are shown in Figure 2 and Figures S9–S11. The rings of the biphenyl unit in [Co(**2**)_2_(NCS)_2_]*_n_*·4.3*n*CHCl_3_ were disordered and were modelled over two positions of fractional occupancies 0.5/0.5 (for the ring containing C15) and 0.6/0.4 (for the terminal ring). In each structure, the Co atom is octahedrally sited with a *trans*-arrangement of thiocyanato ligands, and the 3,2’:6’,3”-tpy domain adopts conformation **A** (Scheme 2). Bond lengths in the cobalt(II) coordination spheres are given in Table 1. Despite significant variation in the angles between the planes of the aromatic rings in coordinated ligands **1**, **2** and **3** (Table 1), the structures of the asymmetric units in [Co(**1**)_2_(NCS)_2_]*_n_*·4.5*n*CHCl_3_ (Figure 2a) and [Co(**2**)_2_(NCS)_2_]*_n_*·4.3*n*CHCl_3_ (Figure 2b) are very similar. The structures propagate into 2-dimensional (4,4) nets with the biphenyl units arranged in cones above and below the plane containing the Co atoms. This is closely related to the structures of solvated [CoL_2_(NCS)_2_]*_n_* in which L is 4’-(4-ethoxyphenyl)-3,2’:6’,3”-tpy, 4’-(4-*n*-propoxyphenyl)-3,2’:6’,3”-tpy, or 4’-(4-*n*-butyloxyphenyl)-3,2’:6’,3”-tpy; these crystallize in the tetragonal space groups *P*4*/ncc* or *P–*42_1_*c* [10]. The cone assemblies in [Co(**1**)_2_(NCS)_2_]*_n_*·4.5*n*CHCl_3_ are shown in Figure 3a, and their arrangement in [Co(**2**)_2_(NCS)_2_]*_n_*·4.3*n*CHCl_3_ is essentially the same (Figure S12). We therefore focus only on the structure of [Co(**1**)_2_(NCS)_2_]*_n_*·4.5*n*CHCl_3_. Each cone is built up around a 4-fold axis, and thus, in Figure 3b, the cones protrude from the square rhombi. [Co(**3**)_2_(NCS)_2_]*_n_*·4*n*CHCl_3_ comprises a 2-dimensional (4,4) net similar to those in [Co(**1**)_2_(NCS)_2_]*_n_*·4.5*n*CHCl_3_ and [Co(**2**)_2_(NCS)_2_]*_n_*·4.3*n*CHCl_3_, although the lower symmetry of the *Pccn* space group with respect to *P*4/*ncc* means that there are two independent terpyridine ligands in [Co(**2**)_2_(NCS)_2_]*_n_*·4.3*n*CHCl_3_ (Figure 2 and Table 1) and the cone assembly is built up around a 2-fold axis (Figure 3c,d).

**Figure 4:**
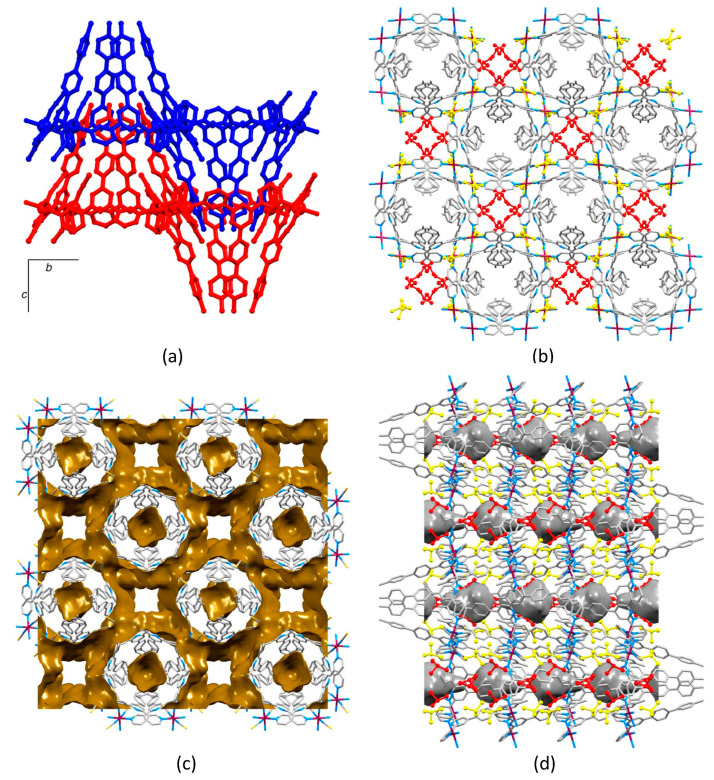
[Co(**1**)_2_(NCS)_2_]*_n_*·4.5*n*CHCl_3_: (**a**) Stacking of cones of biphenyl substituents between adjacent (4,4) nets (H atoms and CHCl_3_ molecules omitted). (**b**) Part of the lattice viewed down the *c*-axis showing that the solvent molecules that were refined (colored red and yellow) occupy two types of cavities. (**c**) The same part of the lattice as in (**b**) illustrating the voids calculated using a contact surface map with probe radius = 1.2 Å, and drawn using Mercury 2020.1 [23]. (**d**) A view down the *b*-axis showing the refined CHCl_3_ molecules (red and yellow) and the residual voids (see text).

**Figure 5:**
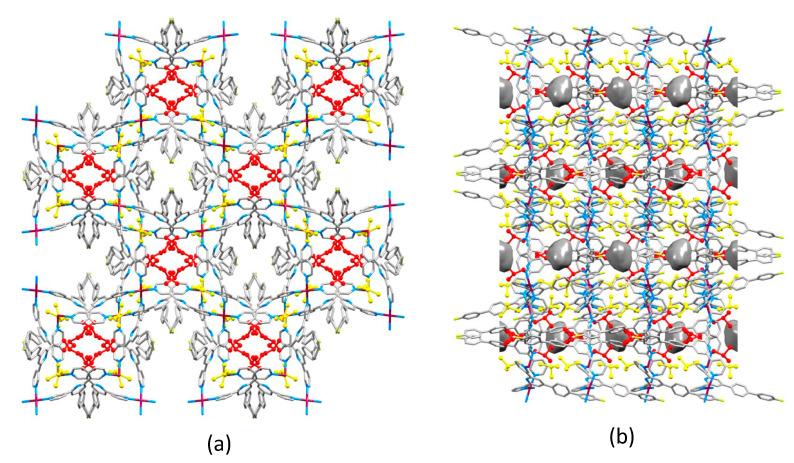
[Co(**3**)_2_(NCS)_2_]*_n_*·4*n*CHCl_3_: (**a**) Part of the lattice viewed down the *c*-axis showing that the solvent molecules (colored red and yellow) occupy two types of cavities. (**b**) The same part of the lattice viewed down the *b*-axis; the residual voids (calculated using a contact surface map with probe radius = 1.2 Å, and drawn using Mercury 2020.1 [23]) that are aligned along the *c*-axis account for 1.4% of the total void.

In each of [Co(**1**)_2_(NCS)_2_]*_n_*·4.5*n*CHCl_3_, [Co(**2**)_2_(NCS)_2_]*_n_*·4.3*n*CHCl_3_, and [Co(**3**)_2_(NCS)_2_]*_n_*·4*n*CHCl_3_, adjacent sheets pack with the cones of biphenyl substituents stacked inside one another along the crystallographic *c*-axis (Figure 4a). Face-to-face π-stacking occurs between the pyridine ring containing N2 (see Figures S9–S11 for atom numbers) in one sheet with the peripheral arene ring in the next sheet. In [Co(**1**)_2_(NCS)_2_]*_n_*·4.5*n*CHCl_3_, the π-stacking interaction is characterized by a centroid distance of 3.75 Å and an angle between the ring planes of 9.6°. These parameters are 3.69 Å and 11.9° in [Co(**2**)_2_(NCS)_2_]*_n_*·4.3*n*CHCl_3_ for one modelled site of the disordered phenyl ring. The cone…cone π-stacking contacts in [Co(**3**)_2_(NCS)_2_]*_n_*·4*n*CHCl_3_ are effective for only one of the two independent ligands with a centroid…centroid distance of 3.63 Å and an inter-ring plane angle of 10.9°. Although the nets and packing of the nets are similar in the compounds with ligands **1**, **2** and **3**, the number of CHCl_3_ molecules per Co differ. Part of the solvent region in each of [Co(**1**)_2_(NCS)_2_]*_n_*·4.5*n*CHCl_3_ and [Co(**2**)_2_(NCS)_2_]*_n_*·4.3*n*CHCl_3_ was treated with a solvent mask because of disordering, and the small difference between the final formulations was carefully checked. There was insufficient residual electron density in the coordination network with **2** to permit a formula of [Co(**2**)_2_(NCS)_2_]*_n_*·4.5*n*CHCl_3_. Further confirmation came from an analysis of the void spaces (calculated in Mercury [23] using a contact surface map with probe radius = 1.2 Å), which revealed 31.7% in [Co(**1**)_2_(NCS)_2_]*_n_* and 29.8% in [Co(**2**)_2_(NCS)_2_]*_n_*. This difference may be attributed to variations in the twist angles between the arene rings of ligands **1** and **2** (Table 1), and the fact that ligand **2** is disordered over two positions. Figure 4b illustrates that the four refined CHCl_3_ molecules (colored red and yellow in Figure 4b) per Co occupy two types of cavities. The solvent accessible voids are illustrated in Figure 4c. Figure 4c was generated in Mercury [23] using a solvent-free lattice, while Figure 4d shows the residual voids after taking into account the refined CHCl_3_ molecules. These essentially closed cavities account for 4.2% of the solvent accessible void in the lattice and accommodate 0.5CHCl_3_ per Co. Figure 5 illustrates the lattice in [Co(**3**)_2_(NCS)_2_]*_n_*·4*n*CHCl_3_. Similarities between Figure 4b and Figure 5a are clear, and the CHCl_3_ molecules are again distributed equally between two types of cavities in the lattice. The remaining closed voids that run along the *c*-axis account for only 1.4% of the total void and host no solvent, in contrast to analogous voids in [Co(**1**)_2_(NCS)_2_]*_n_*·4.5*n*CHCl_3_ and [Co(**2**)_2_(NCS)_2_]*_n_*·4.3*n*CHCl_3_.

### 2.3. Thermogravimetric Analysis (TGA) of [Co(**3**)_2_(NCS)_2_]_n_·4nCHCl_3_

**Figure 6:**
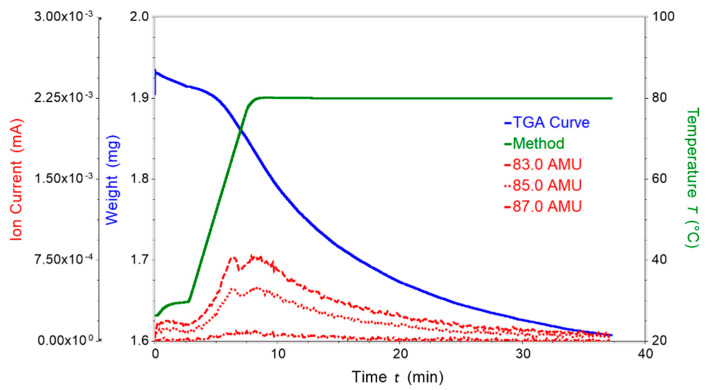
TGA and mass spectrometric traces for the analysis of [Co(**3**)_2_(NCS)_2_]*_n_*·4*n*CHCl_3_ (cycle 1). Green: temperature vs. time; blue: weight of sample vs. time; red: mass detection for *m*/*z* 83.0 (most intense peak), 85.0 and 87.0. The initial mass of sample was 1.93 mg and a weight loss of 0.32 mg corresponds to ca. 17%.

As detailed in Figure 1, PXRD confirmed that the single crystal structure of [Co(**3**)_2_(NCS)_2_]*_n_*·4*n*CHCl_3_ was representative of the bulk material, and therefore this coordination network was selected for an analysis of solvent removal from, and re-entry into, the crystal lattice. Crystals of [Co(**3**)_2_(NCS)_2_]*_n_*·4*n*CHCl_3_ were heated to 80 °C for 30 min. Figure 6 illustrates that loss of CHCl_3_ was detected with mass peaks at *m*/*z* 83.0, 85.0 and 87.0 corresponding to CHCl_2_^+^ as the dominant fragment [24]. In [Co(**3**)_2_(NCS)_2_]*_n_*·4*n*CHCl_3_, four molecules of CHCl_3_ correspond to 32.7% of the molecular weight. The mass loss in the TGA (Figure 6 caption) corresponded to ca. 17%, indicating the loss of two CHCl_3_ per Co atom. This is in accord with the structural data, which revealed that the solvent molecules in [Co(**3**)_2_(NCS)_2_]*_n_*·4*n*CHCl_3_ are equally distributed within two different types of cavities (Figure 5a).

**Figure 7:**
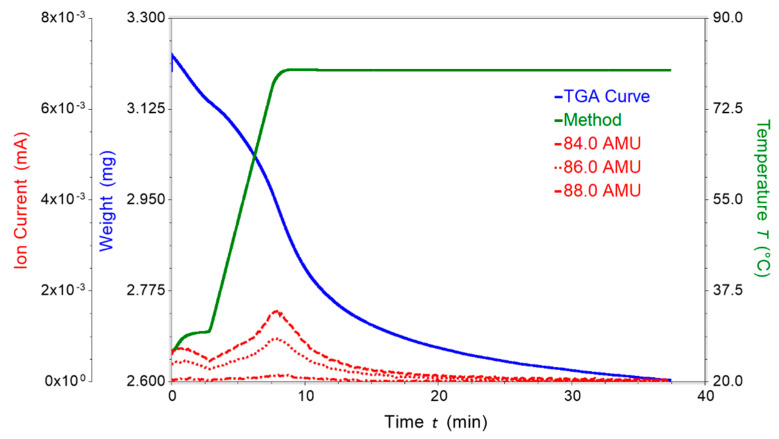
TGA and mass spectrometric traces for the analysis of [Co(**3**)_2_(NCS)_2_]*_n_*·2*n*CHCl_3_·2*n*CDCl_3_ (cycle 3: after 24 h exposure to CDCl_3_ vapor). Green: temperature vs. time; blue: weight of sample vs. time; red: mass detection for *m*/*z* 84.0 (most intense peak), 86.0 and 88.0. The initial mass of sample was 3.22 mg and a weight loss of 0.62 mg corresponds to ca. 19%.

After the initial TGA cycle, the sample was cooled to room temperature and was exposed to CHCl_3_ vapor for 24 h. TGA analysis was repeated (cycle 2) and loss of CHCl_3_ was again detected as shown in Figure S13 in the Supporting Material. A weight loss corresponding to ca. 19% was similar to that in cycle 1. In order to distinguish between the residual lattice solvent after cycle 1, and solvent re-entering the lattice during exposure to CHCl_3_ vapor, a third cycle was carried out using CDCl_3_. The same crystalline material was placed in contact to CDCl_3_ vapor for 24 h and then analyzed by TGA (Figure 7). Mass peaks at *m*/*z* 84.0, 86.0 and 88.0 were detected at ca. 80 °C and were assigned to the CDCl_2_^+^ ion, and the ca. 19% weight loss (see caption to Figure 7) was consistent with the loss of ca. 2 molecules of CDCl_3_ per Co atom. In turn, this is consistent with a formulation of [Co(**3**)_2_(NCS)_2_]*_n_*·2*n*CHCl_3_·2*n*CDCl_3_ after exposure to CDCl_3_ vapor and before TGA cycle 3. The process was finally repeated using CH_2_Cl_2_ vapor. The product of cycle 3, [Co(**3**)_2_(NCS)_2_]*_n_*·2*n*CHCl_3_, was placed in contact with CH_2_Cl_2_ vapor for 24 h. TGA and mass spectrometric analysis of this material showed mass peaks at *m*/*z* 49.0, 51.0, 84.0 and 86.0 arising from CH_2_Cl^+^ and CH_2_Cl_2_^+^ at ca. 40 °C (Figure S14), and the 12% weight loss was consistent with the removal of two molecules of CH_2_Cl_2_ per Co atom.

The results of the TGA experiments illustrate that the coordination network in [Co(**3**)_2_(NCS)_2_]*_n_*·4*n*CHCl_3_ is sufficiently robust to allow half of the solvent to be reversibly removed. Inspection of Figure 4b,c indicates that these are most likely the molecules accommodated in the open channels, which run along the *c*-axis adjacent to the stacked cone-assemblies.

### 2.4. Crystal Structures of [Co(**4**)_2_(NCS)_2_]_n_ and [Co(**5**)_2_(NCS)_2_]_n_·nCHCl_3_

**Figure 8:**
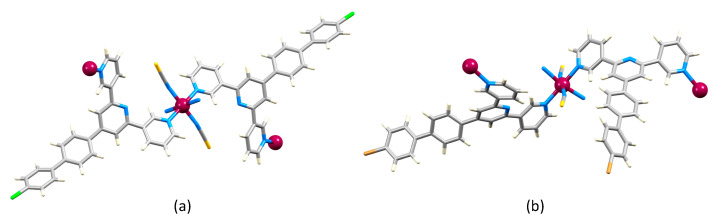
Structures of the asymmetric units in (**a**) [Co(**4**)_2_(NCS)_2_]*_n_* and (**b**) [Co(**5**)_2_(NCS)_2_]*_n_*·*n*CHCl_3_ with symmetry related atoms. [Co(**5**)_2_(NCS)_2_]*_n_*·*n*CHCl_3_ contains two independent ligands. Solvent molecules are omitted. For atom numbering, see Figures S15 and S16. Co: maroon; N: blue; S: yellow; Cl: green; Br: beige.

The compounds [Co(**4**)_2_(NCS)_2_]*_n_* and [Co(**5**)_2_(NCS)_2_]*_n_*·*n*CHCl_3_ both crystallize in the monoclinic space group *P*2_1_/*n*. The structures of the asymmetric units showing the atom numbering are displayed in Figures S15 and S16 in the Supporting Material. In each, the octahedral Co(II) center exhibits a typical *trans*-arrangement of thiocyanato ligands. In [Co(**4**)_2_(NCS)_2_]*_n_*, the Co atom lies on an inversion center (Figure 8a), while [Co(**5**)_2_(NCS)_2_]*_n_*·*n*CHCl_3_ contains two independent 3,2’:6’,3”-terpyridine ligands (Figure 8b). Relevant bond lengths and twist angles between arene rings are presented in Table 2. The units shown in Figure 8 propagate into 2-dimensional (4,4) nets, consistent with the coordination networks assembled with ligands **1**, **2** and **3**,. However, whereas the 3,2’:6’,3”-tpy domains in coordinated **1**–**3** exhibit conformation **A**, that in **4** possesses conformation **B** (Scheme 2 and Figure 8a), while the two independent ligands in [Co(**5**)_2_(NCS)_2_]*_n_*·*n*CHCl_3_ adopt conformations **A** and **B**, respectively (Figure 8b). The two structures are consequently distinct from one another, and from those with ligands **1**–**3**.

**Figure 9:**
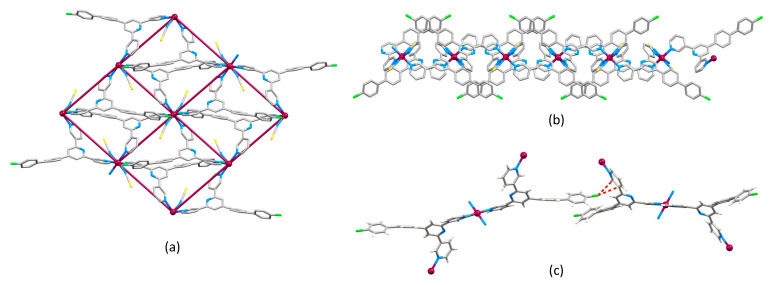
The structure of [Co(**4**)_2_(NCS)_2_]*_n_*. (**a**) Part of the 2D-net showing that the ligands lie over the rhombi, and (**b**) a side view of part of one sheet. The peripheral Cl atoms engage in Cl…π interactions shown in (**c**) with arene rings in the adjacent sheet.

In the (4,4) net in [Co(**4**)_2_(NCS)_2_], the Co atoms lie in a plane, and crystallographic symmetry dictates that all rhombi are identical with internal angles of 82.4° and 97.6°, and the 4’-chloro-[1,1’-biphenyl]-4-yl units are directed up/up/down/down around each rhombus. This contrasts with the cone-assemblies in the compounds containing **1**–**3**. A consequence of the conformational switch of the 3,2’:6’,3”-tpy on going from **1**–**3** to **4** is that the 4’-chloro-[1,1’-biphenyl]-4-yl domains of the ligands lie over the rhombi (Figure 9a,b) rather than projecting directly above the plane. The Cl atoms decorate the outer surfaces of the 2-dimensional sheet in [Co(**4**)_2_(NCS)_2_], and each Cl engages in a Cl…π interaction with a pyridine ring in the adjacent sheet. The shortest contacts are Cl1…C10^vi^ = 3.422(2) and Cl1…C11^vi^ = 3.383(2) Å (symmetry code vi = ^1^/_2_+*x*, ^3^/_2_–*y, –*^1^/_2_+*z*). These distances are within the cut-off value of 3.62 Å applied by Prasanna and Row [25], and the interaction in [Co(**4**)_2_(NCS)_2_], which involves the Cl atom directed at a specific arene π-bond, is classified as semi-localized [26,27]. In contrast to the coordination networks incorporating ligands **1**, **2**, **3** and **5**, that with ligand **4** contains no solvent of crystallization; the void (calculated using a contact surface map with probe radius = 1.2 Å) is <2%. We note that the network contains no face-to-face π-stacking interactions.

**Figure 10:**
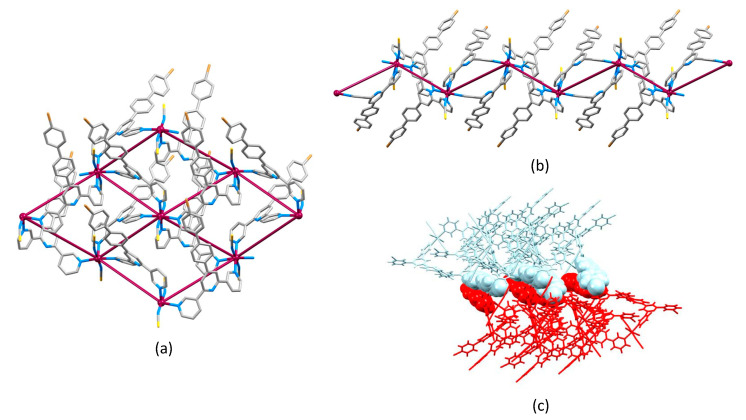
The structure of [Co(**5**)_2_(NCS)_2_]*_n_*·*n*CHCl_3_. (**a**) Part of one 2D-sheet, and (**b**) a side view of the sheet showing the corrugated profile with 4’-bromo-[1,1’-biphenyl]-4-yl substituents protruding above and below the sheet. (**c**) The 4’-bromo-[1,1’-biphenyl]-4-yl substituents in adjacent sheets are closely associated but not π-stacked.

In contrast to the structures described above, the (4,4) net in [Co(**5**)_2_(NCS)_2_]*_n_*·*n*CHCl_3_ (Figure 10a) has a corrugated profile (Figure 10b) with each rhombus adopting a folded conformation with an internal dihedral angle of 124.3°. Of the two independent terpyridine ligands, that with conformation **A** has greater twist angles between the pyridine rings (38.8° and 48.3°) than that with conformation **B** (29.1° and 21.1°). The orientations of the ligands with respect to the (4,4) net defined by the Co atoms is shown in Figure 10b. The 4’-bromo-[1,1’-biphenyl]-4-yl substituents in adjacent sheets are closely associated (Figure 10c) but there are no π-stacking interactions, nor Br…π interactions. The CHCl_3_ molecules occupy channels that follow the crystallogrphic *b*-axis. The difference between the coordination network on going from chloro-substituted ligand **4** to bromo-substituted **5** is striking, but the origins behind the change are unclear. We emphasize that the PXRD (Figure 1b) confirmed that the single crystal structure was representative of the bulk sample.

## 3. Materials and Methods

### 3.1. General

A PerkinElmer UATR Two instrument (Perkin Elmer, 8603 Schwerzenbach, Switzerland) was used to record FT-infrared (IR) spectra. Cobalt(II) thiocyanate was bought from Alfa Aesar and used as received. Compounds **1**–**5** were prepared and characterized as previously described [12].

All crystal growth experiments were carried out under ambient conditions using identical crystallization tubes (i.d. = 13.6 mm, 24 mL).

Thermogravimetric analysis (TGA) was carried out under nitrogen using a TGA5500 instrument (TA Instruments, New Castle, DE 19720, USA) coupled to a Discovery II MS, Cirrus 3, Mass Spectrometer, DMS (TA Instruments, New Castle, DE 19720, USA). A Barchart scanning method in the mass range 10–125 was applied. For each experiment, the temperature of the TGA instrument was initially stabilized at 30 °C, and then the samples were heated to 80 °C and maintained at this temperature for 30 min. The nature of the solvent lost was identified by mass spectrometry. Afterwards the sample was cooled to room temperature (ca. 22 °C) and placed in contact with vapors of CHCl_3_, CDCl_3_ or CH_2_Cl_2_ for 24 h. TGA was then repeated using the above procedure.

### 3.2. [Co(**1**)_2_(NCS)_2_]_n_·4.5nCHCl_3_

A solution of Co(NCS)_2_ (5.3 mg, 0.030 mmol) in MeOH (5 mL) was layered over a CHCl_3_ solution (4 mL) of **1** (11.9 mg, 0.030 mmol). Pink block-like crystals grew within two weeks. A single crystal was selected for X-ray diffraction and the remaining crystals were washed with MeOH and CHCl_3_, dried under vacuum and analyzed by PXRD and FT-IR spectroscopy.

### 3.3. [Co(**2**)_2_(NCS)_2_]_n_·4.3nCHCl_3_

A solution of Co(NCS)_2_ (5.3 mg, 0.030 mmol) in MeOH (5 mL) was layered over a CHCl_3_ solution (5 mL) of **2** (11.6 mg, 0.030 mmol). Pink plate-like crystals grew within two weeks. A single crystal was selected for X-ray diffraction and the remaining crystals were washed with MeOH and CHCl_3_, dried under vacuum and analyzed by PXRD and FT-IR spectroscopy.

### 3.4. [Co(**3**)_2_(NCS)_2_]_n_·4nCHCl_3_

A solution of Co(NCS)_2_ (5.3 mg, 0.030 mmol) in MeOH (5 mL) was layered over a CHCl_3_ solution (4 mL) of **3** (12.1 mg, 0.030 mmol). Pale pink plate-like crystals grew after 1 day. A single crystal was selected for X-ray diffraction and the remaining crystals were washed with MeOH and CHCl_3_, dried under vacuum and analyzed by PXRD and FT-IR spectroscopy.

### 3.5. [Co(**4**)_2_(NCS)_2_]_n_

A solution of Co(NCS)_2_ (5.3 mg, 0.030 mmol) in MeOH (6 mL) was layered over a CHCl_3_ solution (6 mL) of **4** (12.6 mg, 0.030 mmol). Pink block-like crystals grew after 5 days. A single crystal was selected for X-ray diffraction and the remaining crystals were washed with MeOH and CHCl_3_, dried under vacuum and analyzed by PXRD and FT-IR spectroscopy.

### 3.6. [Co(**5**)_2_(NCS)_2_]_n_·nCHCl_3_

A solution of Co(NCS)_2_ (5.3 mg, 0.030 mmol) in MeOH (5 mL) was layered over a CHCl_3_ solution (4 mL) of **5** (13.9 mg, 0.030 mmol). Light pink plate-like crystals grew within two weeks. A single crystal was selected for X-ray diffraction and the remaining crystals were washed with MeOH and CHCl_3_, dried under vacuum and analyzed by PXRD and FT-IR spectroscopy.

### 3.7. Crystallography

Single crystal data were collected on a Bruker APEX-II diffractometer (Bruker AG, 8117 Fällanden, Switzerland) (CuKα radiation) with data reduction, solution and refinement using the programs APEX [28], ShelXT [29], Olex2 [30] and ShelXL v. 2014/7 [31], or using a STOE StadiVari diffractometer equipped with a Pilatus300K detector and with a Metaljet D2 source (GaKα radiation). For the latter, data processing used STOE software (X-Area 1.90, STOE, 2020), and the structure was solved using Superflip [32,33] and Olex2 [30]. The model was refined with ShelXL v. 2014/7 [31]. See Section 3.8, Section 3.9, Section 3.10, Section 3.10, Section 3.11 and Section 3.12 for the radiation type (Cu or Ga). All H atoms were included at geometrically calculated positions and refined using a riding model with U_iso_ = 1.2 of the parent atom. Structure analysis and structural diagrams used CSD Mercury 2020.1 [23].

In [Co(**1**)_2_(NCS)_2_]*_n_*·4.5*n*CHCl_3_ and in [Co(**2**)_2_(NCS)_2_]*_n_*·4.3*n*CHCl_3_, part of the solvent region was treated with a solvent mask, and the electron density removed corresponded to 0.5 and to 2.3 chloroform molecules, respectively. The remaining solvent molecules were refined isotropically.

PXRD patterns were collected at room temperature in transmission mode using a Stoe Stadi P diffractometer (STOE & Cie GmbH, 64295 Darmstadt, Germany) with Cu Kα1 radiation (Ge(111) monochromator) and a DECTRIS MYTHEN 1K detector. Whole-pattern decomposition (profile matching) analysis [34,35,36] of the diffraction patterns was performed with the package FULLPROF SUITE [36,37] (v. September 2020) using a previously determined instrument resolution function based on a NIST640d standard. The structural models were taken from the single crystal X-ray diffraction refinements. Refined parameters in Rietveld were: scale factor, zero shift, lattice parameters, Co and S atomic σ positions, background points and peaks shapes as a Thompson–Cox–Hastings pseudo-Voigt function. Preferred orientations were included in the analysis as a March–Dollase multi-axial phenomenological model.

### 3.8. [Co(**1**)_2_(NCS)_2_]_n_·4.5nCHCl_3_

C_62.50_H_46.50_Cl_13.50_CoN_8_S_2_, *M*_r_ = 1511.20, pink block, tetragonal, space group *P*4*/ncc*, *a* = 26.1874(12), *b* = 26.1874(12), *c* = 19.0189(10) Å, *V* = 13042.8(14) Å^3^, *D*_c_ = 1.539 g cm^–3^, *T* = 150 K, *Z* = 8, μ(Cu*Kα*) = 8.134 mm^−1^. Total 106826 reflections, 5933 unique (*R_int_* = 0.0511). Refinement of 5227 reflections (385 parameters) with *I* > 2*σ*(*I*) converged at final *R*_1_ = 0.0949 (*R*_1_ all data = 0.1027), *wR*_2_ = 0.2926 (*wR*_2_ all data = 0.3016), gof = 1.075. CCDC 2111729.

### 3.9. [Co(**2**)_2_(NCS)_2_]_n_·4.3nCHCl_3_

C_60.3_Cl_12.9_CoH_42.3_N_8_S_2_, *M*_r_ = 1459.27, pink plate, tetragonal, space group *P*4*/ncc*, *a* = 25.8136(8), *b* = 25.8136(8), *c* = 18.8776(6) Å, *V* = 12578.9(9) Å^3^, *D*_c_ = 1.541 g cm^–3^, *T* = 150 K, *Z* = 8, μ(Cu*Kα*) = 8.183 mm^−1^. Total 63834 reflections, 5852 unique (*R_int_* = 0.1506). Refinement of 5020 reflections (318 parameters) with *I* > 2*σ*(*I*) converged at final *R*_1_ = 0.1858 (*R*_1_ all data = 0.1963), *wR*_2_ = 0.4219 (*wR*_2_ all data = 0.4271), gof = 1.125. CCDC 2111727.

### 3.10. [Co(**3**)_2_(NCS)_2_]_n_·4nCHCl_3_

C_60_H_40_Cl_12_CoF_2_N_8_S_2_, *M*_r_ = 1459.45, light pink plate, orthorhombic, space group *Pccn*, *a* = 25.5390(17), *b* = 26.0947(18), *c* = 18.6994(12) Å, *V* = 12461.9(14) Å^3^, *D*_c_ = 1.556 g cm^–3^, *T* = 150 K, *Z* = 8, μ(Cu*Kα*) = 7.962 mm^−1^. Total 147,886 reflections, 11,731 unique (*R_int_* = 0.0739). Refinement of 9492 reflections (766 parameters) with *I* > 2*σ*(*I*) converged at final *R*_1_ = 0.1449 (*R*_1_ all data = 0.1556), *wR*_2_ = 0.4596 (*wR*_2_ all data = 0.4825), gof = 0.979. CCDC 2111726.

### 3.11. [Co(**4**)_2_(NCS)_2_]_n_

C_56_H_36_Cl_2_CoN_8_S_2_, *M*_r_ = 1014.88, pink block, monoclinic, space group *P*2_1_/*n*, *a* = 9.96970(10), *b* = 17.2090(2), *c* = 14.3819(2) Å, *β* = 105.999(2)°, *V* = 2371.91(5) Å^3^, *D*_c_ = 1.421 g cm^–3^, *T* = 150 K, *Z* = 2, μ(Ga*Kα*) = 3.411 mm^−1^. Total 46926 reflections, 5023 unique (*R_int_* = 0.1144). Refinement of 4752 reflections (313 parameters) with *I* > 2*σ*(*I*) converged at final *R*_1_ = 0.0507 (*R*_1_ all data = 0.0527), *wR*_2_ = 0.1383 (*wR*_2_ all data = 0.1399), gof = 1.050. CCDC 2111724.

### 3.12. [Co(**5**)_2_(NCS)_2_]_n_·nCHCl_3_

C_57_H_37_Br_2_Cl_3_CoN_8_S_2_, *M*_r_ = 1223.17, light pink plate, monoclinic, space group *P*2_1_/*n*, *a* = 17.1095(12), *b* = 10.7555(7), *c* = 28.8062(19) Å, *β* = 91.434(3)^o^, *V* = 5299.3(6) Å^3^, *D*_c_ = 1.533 g cm^–3^, *T* = 150 K, *Z* = 4, μ(Cu*Kα*) = 6.808 mm^−1^. Total 70610 reflections, 9840 unique (*R_int_* = 0.0370). Refinement of 9183 reflections (658 parameters) with *I* > 2*σ*(*I*) converged at final *R*_1_ = 0.0453 (*R*_1_ all data = 0.0483), *wR*_2_ = 0.1140 (*wR*_2_ all data = 0.1163), gof = 1.051. CCDC 2111731.

## 4. Conclusions

We have reported the assemblies of five coordination networks from reactions between Co(NCS)_2_ and the 3,2’:6’,3”-tpy ligands **1**–**5**. Each ligand contains a 4’-substituted-[1,1’-biphenyl]-4-yl group in which the substituent is Me (ligand **1**), H (**2**), F (**3**), Cl (**4**) or Br (**5**). [Co(**1**)_2_(NCS)_2_]*_n_*·4.5*n*CHCl_3_, [Co(**2**)_2_(NCS)_2_]*_n_*·4.3*n*CHCl_3_, [Co(**3**)_2_(NCS)_2_]*_n_*·4*n*CHCl_3_, [Co(**4**)_2_(NCS)_2_]*_n_*, and [Co(**5**)_2_(NCS)_2_]*_n_*·*n*CHCl_3_ all consist of 2D-networks in which the Co atoms are 4-connecting nodes. Three structure types are identified. For ligands **1**, **2** and **3**, highly symmetrical nets are formed in which the 3,2’:6’,3”-tpy adopts conformation **A**, and the 4’-substituted-[1,1’-biphenyl]-4-yl units are arranged in cones pointing above and below each 2D-sheet; cone…cone π-stacking interactions contribute to the packing of sheets. The networks are porous, with CHCl_3_ molecules occupying distinct types of cavities which can be differentiated using TGA; half of the solvent in [Co(**3**)_2_(NCS)_2_]*_n_*·4*n*CHCl_3_ can be removed at 80 °C and be replaced by CHCl_3_, CDCl_3_ or CH_2_Cl_2_.

A second structure-type is observed for [Co(**4**)_2_(NCS)_2_]*_n_* in which the organic linkers lie over the rhombi in the planar (4,4) net defined by the Co atoms; the 3,2’:6’,3”-tpy exhibits conformation **B**. Each Cl atom is involved in a Cl…π interaction with a pyridine ring in the adjacent sheet. The network in [Co(**5**)_2_(NCS)_2_]*_n_*·*n*CHCl_3_ represents a third structure-type in which the (4,4) defined by the Co atoms is non-planar, and two independent 3,2’:6’,3”-tpy ligands adopt conformations **A** and **B**, respectively. Although adjacent sheets in [Co(**5**)_2_(NCS)_2_]*_n_*·*n*CHCl_3_ are closely associated, there are no Br…π interactions. Face-to-face π-stacking interactions are not observed in either [Co(**4**)_2_(NCS)_2_]*_n_* or [Co(**5**)_2_(NCS)_2_]*_n_*·*n*CHCl_3_.

This investigation demonstrates that a combination of the conformational flexibility of the 3,2’:6’,3”-tpy metal-binding domain with a change in the peripheral group (Me, H, F, Cl or Br) leads to significant structural variation while retaining a 2D-network defined by 4-connecting Co nodes. With ligands **1**, **2** and **3**, pyridine…arene π-stacking is dominant, whereas Cl…π interactions are important for packing in [Co(**4**)_2_(NCS)_2_]*_n_.* The introduction of the Br substituent in ligand **5** switches off both face-to-face π-stacking and halogen…π-interactions, and the packing interactions are more subtly controlled.

## Figures and Tables

**Table 1:** Selected bond lengths and inter-ring angles in the cobalt(II) coordination polymers containing ligands **1**–**3**.

	Co–N_NCS_/Å	Co–N_tpy_/Å	Angle between Planes of Pyridine Rings/°	Angle between Planes of Pyridine and Arene Rings/°	Angle between Planes of Rings in Biphenyl Unit/°
[Co(**1**)_2_(NCS)_2_]*_n_*·4.5*n*CHCl_3_	2.066(4)	2.211(4), 2.189(4)	27.7, 28.4	17.5	55.6
[Co(**2**)_2_(NCS)_2_]*_n_*·4.3*n*CHCl_3_	2.067(9)	2.166(11), 2.193(10)	24.7, 35.7	35.6 ^a^	79.7 ^a^
[Co(**3**)_2_(NCS)_2_]*_n_*·4*n*CHCl_3_	2.064(7), 2.065(6)	2.164(6), 2.192(6), 2.189(6), 2.213(6)	28.3, 33.3 ^b^ 24.8, 37.1 ^c^	40.2 ^b^ 15.9 ^c^	48.0 ^b^ 58.2 ^c^

^a^ For one orientation of each disordered ring; ^b^ For one of two independent ligands; ^c^ For the second independent ligand.

**Table 2:** Co–N bond lengths and inter-ring angles in the cobalt(II) coordination polymers containing ligands **4** and **5**.

	Co–N_NCS_/Å	Co–N_tpy_/Å	Angle between Planes of Pyridine Rings/°	Angle between Planes of Pyridine and Arene Rings/°	Angle between Planes of Rings in Biphenyl Unit/°
[Co(**4**)_2_(NCS)_2_]*_n_*	2.0776(17)	2.1781(17), 2.2295(18)	34.1, 39.0	41.4	28.8
[Co(**5**)_2_(NCS)_2_]*_n_*·*n*CHCl_3_	2.061(2), 2.084(2)	2.170(2), 2.221(2), 2.175(2), 2.199(2)	29.1, 21.1 ^a^ 38.8, 48.3 ^b^	38.9 ^a^ 22.1 ^b^	40.9 ^a^ 30.6 ^b^

^a^ For one of two independent ligands with conformation **B**; ^b^ for the independent ligand with conformation **A**.

## Data Availability

The data presented in this study are available on request from the corresponding author. The data are not publicly accessible at present.

## Supplementary Materials

The following are available online. Figures S1–S5: IR spectra; Figures S6–S8: PXRD; Figures S9–S12: additional structural figures; Figures S13 and S14: TGA analyses; Figures S15 and S16: additional structural figures.

## Sample Availability

Samples may be made available from the authors upon request.
