# Supplementary material for: Adapting (4,4) Networks through Substituent Effects and Conformationally Flexible 3,2’:6’,3”-Terpyridines
Source: Molecules. 2021 Oct 20;26(21):6337. doi: 10.3390/molecules26216337 (PMC8587907; doi:10.3390/molecules26216337)

checkCIF/PLATON report

Structure factors have been supplied for datablock(s) rod140_150k_

THIS REPORT IS FOR GUIDANCE ONLY. IF USED AS PART OF A REVIEW PROCEDURE FOR PUBLICATION, IT SHOULD NOT REPLACE THE EXPERTISE OF AN EXPERIENCED CRYSTALLOGRAPHIC REFEREE.

No syntax errors found. CIF dictionary Interpreting this report

Datablock: rod140_150k_

```
Bond precision:  C-C = 0.0031 A          Wavelength=1.34050

Cell:            a=9.9697(1)     b=17.2090(2)        c=14.3819(2)
                 alpha=90        beta=105.999(2)     gamma=90
Temperature:     150 K

                Calculated                 Reported
Volume          2371.91(5)                 2371.91(5)
Space group     P 21/n                     P 1 21/n 1
Hall group      -P 2yn                     -P 2yn
Moiety formula  C56 H36 Cl2 Co N8 S2       C56 H36 Cl2 Co N8 S2
Sum formula     C56 H36 Cl2 Co N8 S2       C56 H36 Cl2 Co N8 S2
Mr              1014.88                    1014.88
Dx,g cm-3       1.421                      1.421
Z               2                          2
Mu (mm-1)       3.467                      3.411
F000            1042.0                     1042.0
F000'           1044.93
h,k,lmax        12,21,18                   12,21,18
Nref            5057                       5023
Tmin,Tmax       0.474,0.560                0.454,1.000
Tmin'           0.358

Correction method= # Reported T Limits: Tmin=0.454 Tmax=1.000
AbsCorr = MULTI-SCAN

Data completeness= 0.993          Theta(max)= 58.159

R(reflections)= 0.0507( 4752)     wR2(reflections)= 0.1399( 5023)

S = 1.047                   Npar= 313
```

The following ALERTS were generated. Each ALERT has the format
test-name_ALERT_alert-type_alert-level.
Click on the hyperlinks for more details of the test.

Alert level C

```
RADNW01_ALERT_1_C  The radiation wavelength lies outside the expected range
            for the supplied radiation type. Expected range 1.34130-1.34150
            Wavelength given =    1.34050
PLAT051_ALERT_1_C Mu(calc) and Mu(CIF) Ratio Differs from 1.0 by .       1.65 %
PLAT911_ALERT_3_C Missing FCF Refl Between Thmin & STh/L=    0.600          2 Report
```

Alert level G

```
ABSMU01_ALERT_1_G  Calculation of _exptl_absorpt_correction_mu
                not performed for this radiation type.
PLAT004_ALERT_5_G Polymeric Structure Found with Maximum Dimension          2 Info
PLAT230_ALERT_2_G Hirshfeld Test Diff for    S1       --C28      .        5.2 s.u.
PLAT794_ALERT_5_G Tentative Bond Valency for Co1       (II)      .       1.85 Info
PLAT912_ALERT_4_G Missing # of FCF Reflections Above STh/L=  0.600         33 Note
PLAT933_ALERT_2_G Number of OMIT Records in Embedded .res File ...          1 Note
PLAT978_ALERT_2_G Number C-C Bonds with Positive Residual Density.         11 Info
PLAT984_ALERT_1_G The  C-f'=    0.0147 Deviates from the B&C-Value     0.0137 Check
PLAT984_ALERT_1_G The Cl-f'=    0.3292 Deviates from the B&C-Value     0.3281 Check
PLAT984_ALERT_1_G The  N-f'=    0.0253 Deviates from the B&C-Value     0.0241 Check
PLAT985_ALERT_1_G The Cl-f"=    0.5397 Deviates from the B&C-Value     0.5435 Check
PLAT985_ALERT_1_G The Co-f"=    2.8797 Deviates from the B&C-Value     2.9049 Check
PLAT985_ALERT_1_G The  S-f"=    0.4242 Deviates from the B&C-Value     0.4295 Check
```

```
   0 ALERT level A = Most likely a serious problem - resolve or explain
   0 ALERT level B = A potentially serious problem, consider carefully
   3 ALERT level C = Check. Ensure it is not caused by an omission or oversight
  13 ALERT level G = General information/check it is not something unexpected

   9 ALERT type 1 CIF construction/syntax error, inconsistent or missing data
   3 ALERT type 2 Indicator that the structure model may be wrong or deficient
   1 ALERT type 3 Indicator that the structure quality may be low
   1 ALERT type 4 Improvement, methodology, query or suggestion
   2 ALERT type 5 Informative message, check
```

It is advisable to attempt to resolve as many as possible of the alerts in all categories. Often the minor alerts point to easily fixed oversights, errors and omissions in your CIF or refinement strategy, so attention to these fine details can be worthwhile. In order to resolve some of the more serious problems it may be necessary to carry out additional measurements or structure refinements. However, the purpose of your study may justify the reported deviations and the more serious of these should normally be commented upon in the discussion or experimental section of a paper or in the "special_details" fields of the CIF. checkCIF was carefully designed to identify outliers and unusual parameters, but every test has its limitations and alerts that are not important in a particular case may appear. Conversely, the absence of alerts does not guarantee there are no aspects of the results needing attention. It is up to the individual to critically assess their own results and, if necessary, seek expert advice.

Publication of your CIF in IUCr journals

A basic structural check has been run on your CIF. These basic checks will be run on all CIFs submitted for publication in IUCr journals (*Acta Crystallographica*, *Journal of Applied Crystallography*, *Journal of Synchrotron Radiation*); however, if you intend to submit to *Acta Crystallographica Section C* or *E* or *IUCrData*, you should make sure that full publication checks are run on the final version of your CIF prior to submission.

Publication of your CIF in other journals

Please refer to the *Notes for Authors* of the relevant journal for any special instructions relating to CIF submission.

PLATON version of 10/08/2020; check.def file version of 06/08/2020

Datablock rod140_150k_ - ellipsoid plot

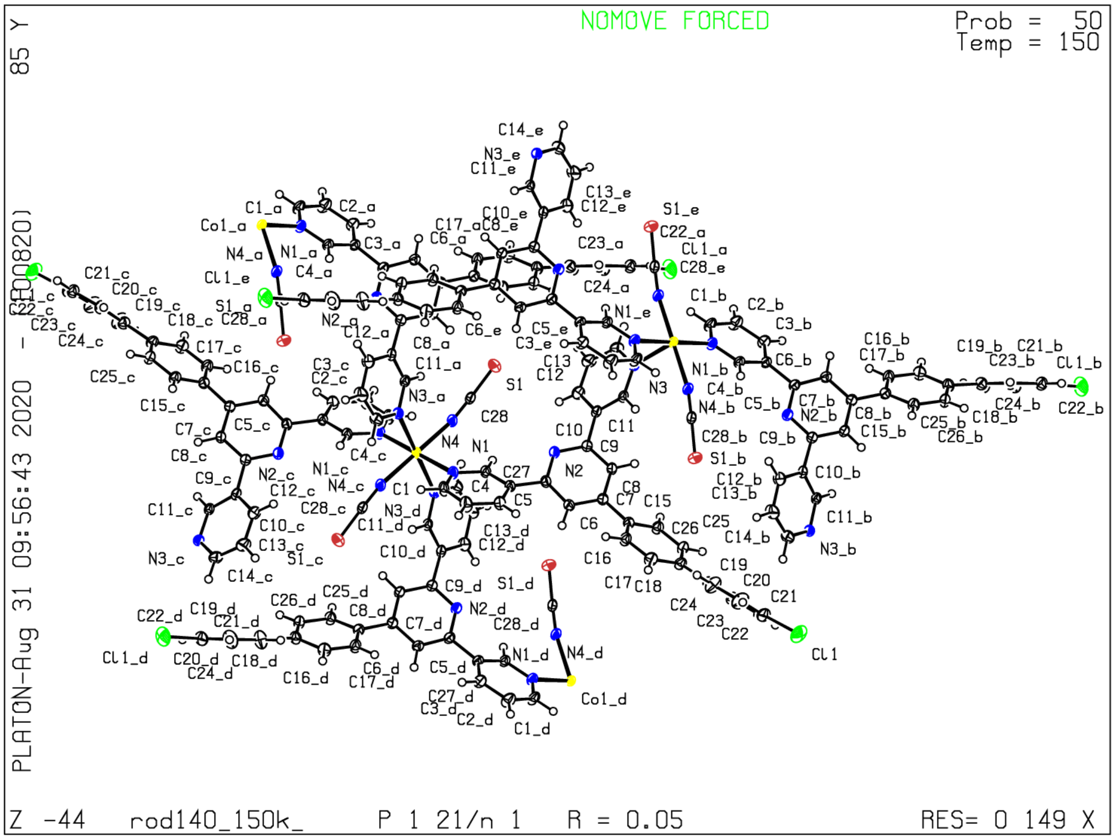

Supplement: Supplementary file 1 [file molecules-26-06337-s001.zip › supporting information-1434316-proof/ROD140_150K__cifreport.pdf]
