# Supplementary material for: Adapting (4,4) Networks through Substituent Effects and Conformationally Flexible 3,2’:6’,3”-Terpyridines
Source: Molecules. 2021 Oct 20;26(21):6337. doi: 10.3390/molecules26216337 (PMC8587907; doi:10.3390/molecules26216337)

checkCIF/PLATON report

Structure factors have been supplied for datablock(s) rod144b_150k_0m

THIS REPORT IS FOR GUIDANCE ONLY. IF USED AS PART OF A REVIEW PROCEDURE FOR PUBLICATION, IT SHOULD NOT REPLACE THE EXPERTISE OF AN EXPERIENCED CRYSTALLOGRAPHIC REFEREE.

No syntax errors found. CIF dictionary Interpreting this report

Datablock: rod144b_150k_0m

Bond precision:	C-C = 0.0043 A		Wavelength=1.54178
Cell:	a=17.1095(12)	b=10.7555(7)	c=28.8062(19)
	alpha=90	beta=91.434(3)	gamma=90
Temperature:	150 K		

	Calculated	Reported
Volume	5299.3(6)	5299.3(6)
Space group	P 21/n	P 1 21/n 1
Hall group	-P 2yn	-P 2yn
Moiety formula	C56 H36 Br2 Co N8 S2, C H Cl3	C56 H36 Br2 Co N8 S2, C H Cl3
Sum formula	C57 H37 Br2 Cl3 Co N8 S2	C57 H37 Br2 Cl3 Co N8 S2
Mr	1223.15	1223.17
Dx,g cm-3	1.533	1.533
Z	4	4
Mu (mm-1)	6.808	6.808
F000	2460.0	2460.0
F000'	2457.16	
h,k,lmax	20,13,35	20,12,34
Nref	10112	9840
Tmin,Tmax	0.244,0.360	0.564,0.753
Tmin'	0.156	

Correction method= # Reported T Limits: Tmin=0.564 Tmax=0.753
AbsCorr = MULTI-SCAN

Data completeness= 0.973 Theta(max)= 70.340

R(reflections)= 0.0453(9183) wR2(reflections)= 0.1163(9840)

S = 1.051 Npar= 658

```
The following ALERTS were generated. Each ALERT has the format
       test-name_ALERT_alert-type_alert-level.
Click on the hyperlinks for more details of the test.
```

Alert level C

```
PLAT244_ALERT_4_C Low    'Solvent' Ueq as Compared to Neighbors of        C57 Check
PLAT601_ALERT_2_C Unit Cell Contains Solvent Accessible VOIDS of .         41 Ang**3
PLAT911_ALERT_3_C Missing FCF Refl Between Thmin & STh/L=    0.600         21 Report
PLAT971_ALERT_2_C Check Calcd Resid. Dens.  1.11A   From Br1             1.55 eA-3
PLAT971_ALERT_2_C Check Calcd Resid. Dens.  1.18A   From Br1             1.54 eA-3
```

Alert level G

```
PLAT004_ALERT_5_G Polymeric Structure Found with Maximum Dimension          2 Info
PLAT083_ALERT_2_G SHELXL Second Parameter in WGHT  Unusually Large      10.77 Why ?
PLAT434_ALERT_2_G Short Inter HL..HL Contact Cl2      ..Cl3              3.12 Ang.
                                       5/2-x,-1/2+y,1/2-z  =      2_745 Check
PLAT794_ALERT_5_G Tentative Bond Valency for Co1       (II)      .       1.89 Info
PLAT910_ALERT_3_G Missing # of FCF Reflection(s) Below Theta(Min).          1 Note
PLAT912_ALERT_4_G Missing # of FCF Reflections Above STh/L=  0.600        224 Note
PLAT978_ALERT_2_G Number C-C Bonds with Positive Residual Density.         11 Info
```

```
   0 ALERT level A = Most likely a serious problem - resolve or explain
   0 ALERT level B = A potentially serious problem, consider carefully
   5 ALERT level C = Check. Ensure it is not caused by an omission or oversight
   7 ALERT level G = General information/check it is not something unexpected

   0 ALERT type 1 CIF construction/syntax error, inconsistent or missing data
   6 ALERT type 2 Indicator that the structure model may be wrong or deficient
   2 ALERT type 3 Indicator that the structure quality may be low
   2 ALERT type 4 Improvement, methodology, query or suggestion
   2 ALERT type 5 Informative message, check
```

Validation response form

Please find below a validation response form (VRF) that can be filled in and pasted into your CIF.

```
# start Validation Reply Form
_vrf_PLAT244_rod144b_150k_0m
;
PROBLEM: Low    'Solvent' Ueq as Compared to Neighbors of        C57 Check
RESPONSE: ...
;
_vrf_PLAT601_rod144b_150k_0m
;
PROBLEM: Unit Cell Contains Solvent Accessible VOIDS of .         41 Ang**3
RESPONSE: ...
;
_vrf_PLAT911_rod144b_150k_0m
;
PROBLEM: Missing FCF Refl Between Thmin & STh/L=    0.600         21 Report
RESPONSE: ...
```

```
;
_vrf_PLAT971_rod144b_150k_0m
;
PROBLEM: Check Calcd Resid. Dens.  1.11A   From Br1            1.55 eA-3
RESPONSE: ...
;
# end Validation Reply Form
```

It is advisable to attempt to resolve as many as possible of the alerts in all categories. Often the minor alerts point to easily fixed oversights, errors and omissions in your CIF or refinement strategy, so attention to these fine details can be worthwhile. In order to resolve some of the more serious problems it may be necessary to carry out additional measurements or structure refinements. However, the purpose of your study may justify the reported deviations and the more serious of these should normally be commented upon in the discussion or experimental section of a paper or in the "special_details" fields of the CIF. checkCIF was carefully designed to identify outliers and unusual parameters, but every test has its limitations and alerts that are not important in a particular case may appear. Conversely, the absence of alerts does not guarantee there are no aspects of the results needing attention. It is up to the individual to critically assess their own results and, if necessary, seek expert advice.

Publication of your CIF in IUCr journals

A basic structural check has been run on your CIF. These basic checks will be run on all CIFs submitted for publication in IUCr journals (*Acta Crystallographica*, *Journal of Applied Crystallography*, *Journal of Synchrotron Radiation*); however, if you intend to submit to *Acta Crystallographica Section C* or *E* or *IUCrData*, you should make sure that full publication checks are run on the final version of your CIF prior to submission.

Publication of your CIF in other journals

Please refer to the *Notes for Authors* of the relevant journal for any special instructions relating to CIF submission.

PLATON version of 13/07/2021; check.def file version of 13/07/2021

Datablock rod144b_150k_0m - ellipsoid plot

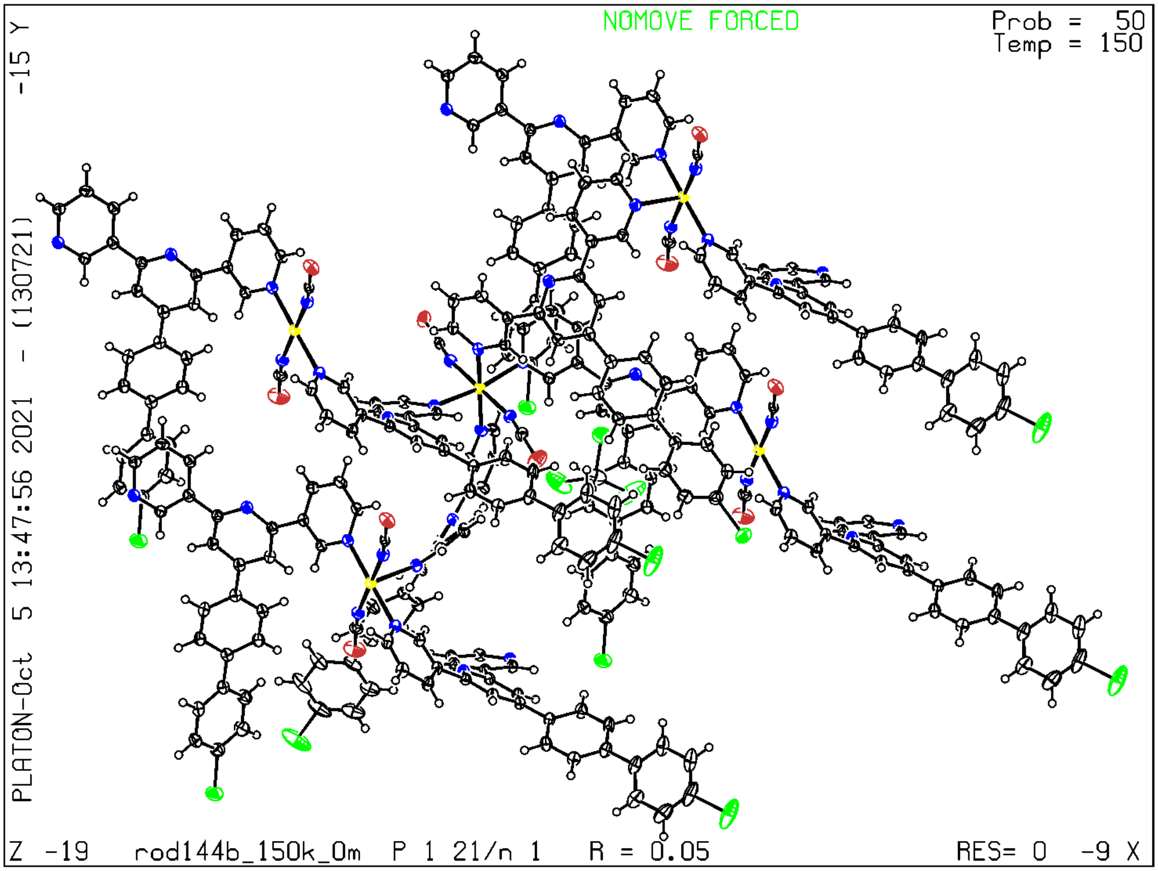

Supplement: Supplementary file 1 [file molecules-26-06337-s001.zip › supporting information-1434316-proof/ROD144B_150K_checkcif.pdf]
