# Supplementary material for: Adapting (4,4) Networks through Substituent Effects and Conformationally Flexible 3,2’:6’,3”-Terpyridines
Source: Molecules. 2021 Oct 20;26(21):6337. doi: 10.3390/molecules26216337 (PMC8587907; doi:10.3390/molecules26216337)

checkCIF/PLATON report

Structure factors have been supplied for datablock(s) rod145_0m

THIS REPORT IS FOR GUIDANCE ONLY. IF USED AS PART OF A REVIEW PROCEDURE FOR PUBLICATION, IT SHOULD NOT REPLACE THE EXPERTISE OF AN EXPERIENCED CRYSTALLOGRAPHIC REFEREE.

No syntax errors found. CIF dictionary Interpreting this report

Datablock: rod145_0m

Bond precision: C-C = 0.0089 A Wavelength=1.54178

Cell:	a=26.1874(12)	b=26.1874(12)	c=19.0189(10)
	alpha=90	beta=90	gamma=90
Temperature:	150 K		

	Calculated	Reported
Volume	13042.8(14)	13042.8(14)
Space group	P 4/n c c	P 4/n c c
Hall group	-P 4a 2ac	-P 4a 2ac
Moiety formula	C58 H42 Co N8 S2, 4(C H Cl3) [+ solvent]	C58 H42 Co N8 S2, 4(C H Cl3), 0.5[CHCL3]
Sum formula	C62 H46 Cl12 Co N8 S2 [+ solvent]	C62.50 H46.50 Cl13.50 Co N8 S2
Mr	1451.52	1511.20
Dx,g cm-3	1.478	1.539
Z	8	8
Mu (mm-1)	7.557	8.134
F000	5896.0	6128.0
F000’	5928.54	
h,k,lmax	31,31,23	31,30,22
Nref	6069	5933
Tmin,Tmax	0.192,0.197	0.611,0.753
Tmin’	0.123	

Correction method= # Reported T Limits: Tmin=0.611 Tmax=0.753
AbsCorr = MULTI-SCAN

Data completeness= 0.978 Theta(max)= 68.911

R(reflections)= 0.0949(5227) wR2(reflections)= 0.3016(5933)

S = 1.075 Npar= 385

The following ALERTS were generated. Each ALERT has the format
test-name_ALERT_alert-type_alert-level.
Click on the hyperlinks for more details of the test.

Alert level B

```
PLAT934_ALERT_3_B Number of (Iobs-Icalc)/Sigma(W) > 10 Outliers ..          2 Check
```

Alert level C

```
DIFMX02_ALERT_1_C  The maximum difference density is > 0.1*ZMAX*0.75
            The relevant atom site should be identified.
PLAT084_ALERT_3_C High wR2 Value (i.e. > 0.25) ...................       0.30 Report
PLAT097_ALERT_2_C Large Reported Max.  (Positive) Residual Density       2.22 eA-3
PLAT244_ALERT_4_C Low    'Solvent' Ueq as Compared to Neighbors of        C31 Check
PLAT341_ALERT_3_C Low Bond Precision on  C-C Bonds ...............     0.0089 Ang.
PLAT906_ALERT_3_C Large K Value in the Analysis of Variance ......      3.469 Check
PLAT911_ALERT_3_C Missing FCF Refl Between Thmin & STh/L=    0.600         12 Report
PLAT918_ALERT_3_C Reflection(s) with I(obs) much Smaller I(calc) .          7 Check
PLAT973_ALERT_2_C Check Calcd Positive Resid. Density on       Co1       1.28 eA-3
```

Alert level G

```
FORMU01_ALERT_2_G  There is a discrepancy between the atom counts in the
            _chemical_formula_sum and the formula from the _atom_site* data.
            Atom count from _chemical_formula_sum:C62.5 H46.5 Cl13.5 Co1 N8 S2
            Atom count from the _atom_site data:  C62 H46 Cl12 Co1 N8 S2
CELLZ01_ALERT_1_G Difference between formula and atom_site contents detected.
CELLZ01_ALERT_1_G ALERT: Large difference may be due to a
            symmetry error - see SYMMG tests
           From the CIF: _cell_formula_units_Z    8
           From the CIF: _chemical_formula_sum  C62.50 H46.50 Cl13.50 Co N8 S2
           TEST: Compare cell contents of formula and atom_site data

           atom    Z*formula  cif sites diff
           C         500.00    496.00    4.00
           H         372.00    368.00    4.00
           Cl        108.00     96.00   12.00
           Co          8.00      8.00    0.00
           N          64.00     64.00    0.00
           S          16.00     16.00    0.00
PLAT004_ALERT_5_G Polymeric Structure Found with Maximum Dimension          2 Info
PLAT041_ALERT_1_G Calc. and Reported SumFormula    Strings  Differ     Please Check
PLAT051_ALERT_1_G Mu(calc) and Mu(CIF) Ratio Differs from 1.0 by .       7.09 %
PLAT068_ALERT_1_G Reported F000 Differs from Calcd (or Missing)...     Please Check
PLAT072_ALERT_2_G SHELXL First  Parameter in WGHT  Unusually Large       0.18 Report
PLAT083_ALERT_2_G SHELXL Second Parameter in WGHT  Unusually Large      70.81 Why ?
PLAT605_ALERT_4_G Largest Solvent Accessible VOID in the Structure        165 A**3
PLAT794_ALERT_5_G Tentative Bond Valency for Co1       (II)      .       1.88 Info
PLAT868_ALERT_4_G ALERTS Due to the Use of _smtbx_masks Suppressed          ! Info
PLAT912_ALERT_4_G Missing # of FCF Reflections Above STh/L=  0.600        125 Note
PLAT978_ALERT_2_G Number C-C Bonds with Positive Residual Density.          5 Info
```

---

0 **ALERT level A** = Most likely a serious problem - resolve or explain
1 **ALERT level B** = A potentially serious problem, consider carefully
9 **ALERT level C** = Check. Ensure it is not caused by an omission or oversight
14 **ALERT level G** = General information/check it is not something unexpected

6 ALERT type 1 CIF construction/syntax error, inconsistent or missing data

```
6 ALERT type 2 Indicator that the structure model may be wrong or deficient
6 ALERT type 3 Indicator that the structure quality may be low
4 ALERT type 4 Improvement, methodology, query or suggestion
2 ALERT type 5 Informative message, check
```

It is advisable to attempt to resolve as many as possible of the alerts in all categories. Often the minor alerts point to easily fixed oversights, errors and omissions in your CIF or refinement strategy, so attention to these fine details can be worthwhile. In order to resolve some of the more serious problems it may be necessary to carry out additional measurements or structure refinements. However, the purpose of your study may justify the reported deviations and the more serious of these should normally be commented upon in the discussion or experimental section of a paper or in the "special_details" fields of the CIF. checkCIF was carefully designed to identify outliers and unusual parameters, but every test has its limitations and alerts that are not important in a particular case may appear. Conversely, the absence of alerts does not guarantee there are no aspects of the results needing attention. It is up to the individual to critically assess their own results and, if necessary, seek expert advice.

Publication of your CIF in IUCr journals

A basic structural check has been run on your CIF. These basic checks will be run on all CIFs submitted for publication in IUCr journals (*Acta Crystallographica*, *Journal of Applied Crystallography*, *Journal of Synchrotron Radiation*); however, if you intend to submit to *Acta Crystallographica Section C* or *E* or *IUCrData*, you should make sure that full publication checks are run on the final version of your CIF prior to submission.

Publication of your CIF in other journals

Please refer to the *Notes for Authors* of the relevant journal for any special instructions relating to CIF submission.

PLATON version of 10/08/2020; check.def file version of 06/08/2020

Datablock rod145_0m - ellipsoid plot

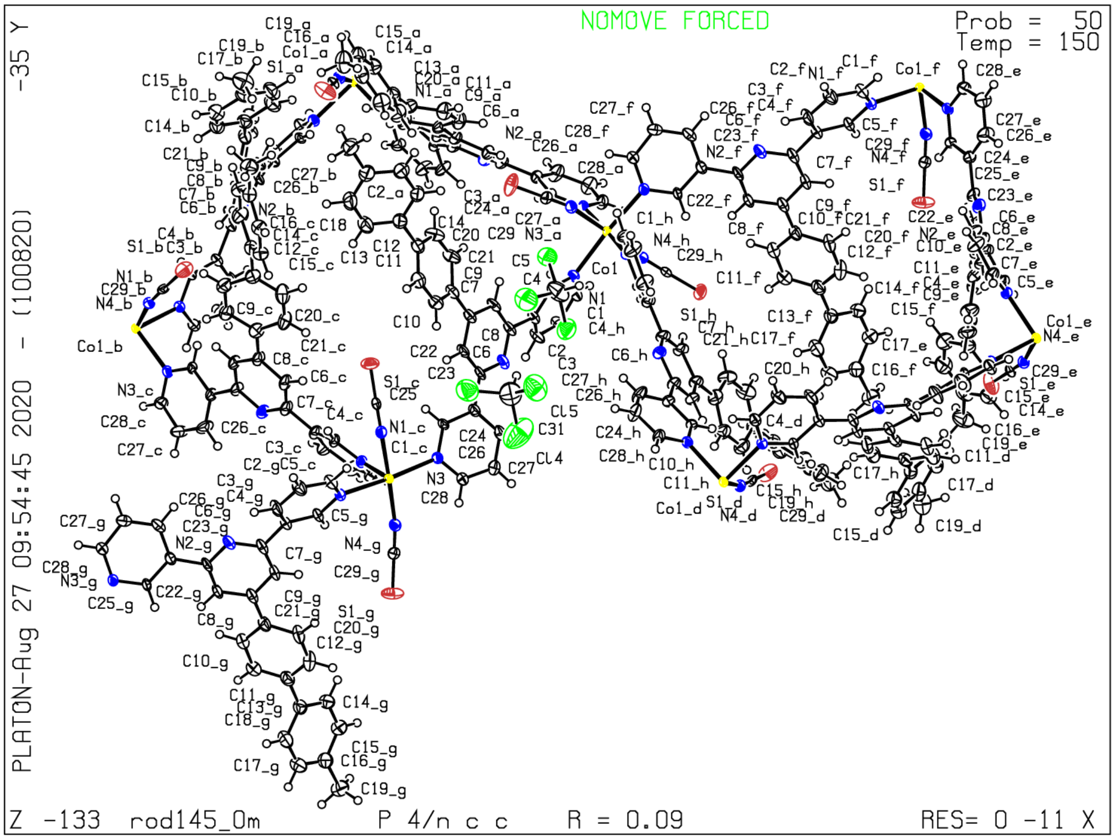

Supplement: Supplementary file 1 [file molecules-26-06337-s001.zip › supporting information-1434316-proof/ROD145_0m_cifreport.pdf]
