# Supplementary material for: Adapting (4,4) Networks through Substituent Effects and Conformationally Flexible 3,2’:6’,3”-Terpyridines
Source: Molecules. 2021 Oct 20;26(21):6337. doi: 10.3390/molecules26216337 (PMC8587907; doi:10.3390/molecules26216337)

checkCIF/PLATON report

Structure factors have been supplied for datablock(s) rod148a_150k

THIS REPORT IS FOR GUIDANCE ONLY. IF USED AS PART OF A REVIEW PROCEDURE FOR PUBLICATION, IT SHOULD NOT REPLACE THE EXPERTISE OF AN EXPERIENCED CRYSTALLOGRAPHIC REFEREE.

No syntax errors found. CIF dictionary Interpreting this report

Datablock: rod148a_150k

Bond precision: C-C = 0.0128 A Wavelength=1.54178

Cell:	a=25.5390(17)	b=26.0947(18)	c=18.6994(12)
	alpha=90	beta=90	gamma=90
Temperature:	150 K		

	Calculated	Reported
Volume	12461.9(14)	12461.9(14)
Space group	P c c n	P c c n
Hall group	-P 2ab 2ac	-P 2ab 2ac
Moiety formula	C56 H36 Co F2 N8 S2, 4(C H Cl3)	C56 H36 Co F2 N8 S2, 4(C H Cl3)
Sum formula	C60 H40 Cl12 Co F2 N8 S2	C60 H40 Cl12 Co F2 N8 S2
Mr	1459.45	1459.45
Dx,g cm-3	1.556	1.556
Z	8	8
Mu (mm-1)	7.962	7.962
F000	5896.0	5896.0
F000’	5929.43	
h,k,lmax	31,31,22	30,31,22
Nref	11963	11731
Tmin,Tmax	0.298,0.727	0.590,0.753
Tmin’	0.191	

Correction method= # Reported T Limits: Tmin=0.590 Tmax=0.753
AbsCorr = MULTI-SCAN

Data completeness= 0.981 Theta(max)= 70.607

R(reflections)= 0.1449(9492) wR2(reflections)= 0.4825(11731)

S = 0.979 Npar= 766

The following ALERTS were generated. Each ALERT has the format
test-name_ALERT_alert-type_alert-level.
Click on the hyperlinks for more details of the test.

Alert level A

```
PLAT084_ALERT_3_A High wR2 Value (i.e. > 0.25) ...................       0.48 Report
```

Author Response: Intensities dropped quite consistently with the resolution. the high resolution area is quite weak and this is probably the cause of this al

---

Alert level B

```
PLAT972_ALERT_2_B Check Calcd Resid. Dens.  0.11A   From Cl12          -3.03 eA-3
PLAT973_ALERT_2_B Check Calcd Positive Resid. Density on       Co1       1.95 eA-3
```

Alert level C

```
DIFMN02_ALERT_2_C  The minimum difference density is < -0.1*ZMAX*0.75
            _refine_diff_density_min given =     -2.631
            Test value =     -2.025
DIFMN03_ALERT_1_C  The minimum difference density is < -0.1*ZMAX*0.75
            The relevant atom site should be identified.
PLAT082_ALERT_2_C High R1 Value .................................       0.14 Report
PLAT098_ALERT_2_C Large Reported Min.  (Negative) Residual Density      -2.63 eA-3
PLAT341_ALERT_3_C Low Bond Precision on  C-C Bonds ...............    0.01275 Ang.
PLAT601_ALERT_2_C Unit Cell Contains Solvent Accessible VOIDS of .         57 Ang**3
PLAT906_ALERT_3_C Large K Value in the Analysis of Variance ......      4.017 Check
PLAT906_ALERT_3_C Large K Value in the Analysis of Variance ......      2.071 Check
PLAT911_ALERT_3_C Missing FCF Refl Between Thmin & STh/L=    0.600         25 Report
PLAT918_ALERT_3_C Reflection(s) with I(obs) much Smaller I(calc) .          1 Check
PLAT971_ALERT_2_C Check Calcd Resid. Dens.  0.85A   From Cl3           1.91 eA-3
PLAT971_ALERT_2_C Check Calcd Resid. Dens.  0.68A   From Cl1           1.90 eA-3
PLAT971_ALERT_2_C Check Calcd Resid. Dens.  1.10A   From Cl5           1.72 eA-3
PLAT971_ALERT_2_C Check Calcd Resid. Dens.  0.94A   From C57           1.66 eA-3
PLAT972_ALERT_2_C Check Calcd Resid. Dens.  0.10A   From Cl8          -2.46 eA-3
PLAT972_ALERT_2_C Check Calcd Resid. Dens.  0.09A   From Cl9          -2.44 eA-3
PLAT972_ALERT_2_C Check Calcd Resid. Dens.  0.18A   From Cl11         -2.44 eA-3
PLAT972_ALERT_2_C Check Calcd Resid. Dens.  0.08A   From Cl7          -2.31 eA-3
PLAT972_ALERT_2_C Check Calcd Resid. Dens.  0.17A   From Cl10         -2.24 eA-3
PLAT972_ALERT_2_C Check Calcd Resid. Dens.  0.05A   From Cl6          -1.70 eA-3
PLAT972_ALERT_2_C Check Calcd Resid. Dens.  0.24A   From Cl4          -1.58 eA-3
PLAT977_ALERT_2_C Check Negative Difference Density on H23            -0.40 eA-3
```

Alert level G

```
PLAT004_ALERT_5_G Polymeric Structure Found with Maximum Dimension          2 Info
PLAT072_ALERT_2_G SHELXL First  Parameter in WGHT  Unusually Large       0.50 Report
PLAT794_ALERT_5_G Tentative Bond Valency for Co1       (II)      .       1.91 Info
PLAT802_ALERT_4_G CIF Input Record(s) with more than 80 Characters          1 Info
PLAT910_ALERT_3_G Missing # of FCF Reflection(s) Below Theta(Min).          1 Note
PLAT912_ALERT_4_G Missing # of FCF Reflections Above STh/L=  0.600        198 Note
PLAT933_ALERT_2_G Number of OMIT Records in Embedded .res File ...         16 Note
PLAT978_ALERT_2_G Number C-C Bonds with Positive Residual Density.          0 Info
```

---

1 **ALERT level A** = Most likely a serious problem - resolve or explain

```
 2 ALERT level B = A potentially serious problem, consider carefully
22 ALERT level C = Check. Ensure it is not caused by an omission or oversight
 8 ALERT level G = General information/check it is not something unexpected

 1 ALERT type 1 CIF construction/syntax error, inconsistent or missing data
21 ALERT type 2 Indicator that the structure model may be wrong or deficient
 7 ALERT type 3 Indicator that the structure quality may be low
 2 ALERT type 4 Improvement, methodology, query or suggestion
 2 ALERT type 5 Informative message, check
```

It is advisable to attempt to resolve as many as possible of the alerts in all categories. Often the minor alerts point to easily fixed oversights, errors and omissions in your CIF or refinement strategy, so attention to these fine details can be worthwhile. In order to resolve some of the more serious problems it may be necessary to carry out additional measurements or structure refinements. However, the purpose of your study may justify the reported deviations and the more serious of these should normally be commented upon in the discussion or experimental section of a paper or in the "special_details" fields of the CIF. checkCIF was carefully designed to identify outliers and unusual parameters, but every test has its limitations and alerts that are not important in a particular case may appear. Conversely, the absence of alerts does not guarantee there are no aspects of the results needing attention. It is up to the individual to critically assess their own results and, if necessary, seek expert advice.

Publication of your CIF in IUCr journals

A basic structural check has been run on your CIF. These basic checks will be run on all CIFs submitted for publication in IUCr journals (*Acta Crystallographica*, *Journal of Applied Crystallography*, *Journal of Synchrotron Radiation*); however, if you intend to submit to *Acta Crystallographica Section C* or *E* or *IUCrData*, you should make sure that full publication checks are run on the final version of your CIF prior to submission.

Publication of your CIF in other journals

Please refer to the *Notes for Authors* of the relevant journal for any special instructions relating to CIF submission.

PLATON version of 10/08/2020; check.def file version of 06/08/2020

Datablock rod148a_150k - ellipsoid plot

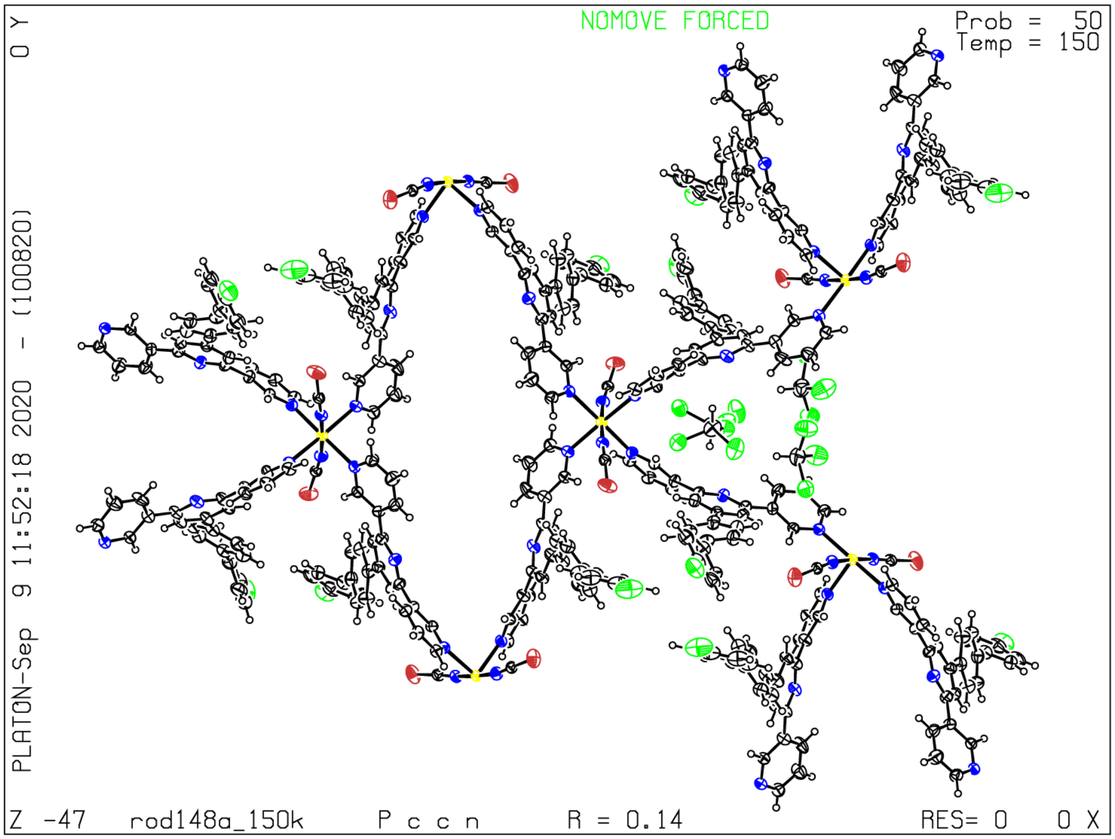

Supplement: Supplementary file 1 [file molecules-26-06337-s001.zip › supporting information-1434316-proof/ROD148A_150K_cifreport.pdf]
