# Supplementary material for: Adapting (4,4) Networks through Substituent Effects and Conformationally Flexible 3,2’:6’,3”-Terpyridines
Source: Molecules. 2021 Oct 20;26(21):6337. doi: 10.3390/molecules26216337 (PMC8587907; doi:10.3390/molecules26216337)

checkCIF/PLATON report

Structure factors have been supplied for datablock(s) rod149c_0m

THIS REPORT IS FOR GUIDANCE ONLY. IF USED AS PART OF A REVIEW PROCEDURE FOR PUBLICATION, IT SHOULD NOT REPLACE THE EXPERTISE OF AN EXPERIENCED CRYSTALLOGRAPHIC REFEREE.

No syntax errors found. CIF dictionary Interpreting this report

Datablock: rod149c_0m

Bond precision:	C-C = 0.0206 A		Wavelength=1.54178
Cell:	a=25.8136(8)	b=25.8136(8)	c=18.8776(6)
	alpha=90	beta=90	gamma=90
Temperature:	150 K		

	Calculated	Reported
Volume	12578.9(9)	12578.9(9)
Space group	P 4/n c c	P 4/n c c
Hall group	-P 4a 2ac	-P 4a 2ac
Moiety formula	C56 H38 Co N8 S2, 4(C H Cl3) [+ solvent]	C56 H38 Co N8 S2, 4(C H Cl3), 0.3[CHCL3]
Sum formula	C60 H42 Cl12 Co N8 S2 [+ solvent]	C60.30 H42.30 Cl12.90 Co N8 S2
Mr	1423.47	1459.27
Dx,g cm-3	1.503	1.541
Z	8	8
Mu (mm-1)	7.824	8.183
F000	5768.0	5907.0
F000’	5800.29	
h,k,lmax	31,31,23	30,30,23
Nref	5976	5852
Tmin,Tmax	0.243,0.441	0.493,0.753
Tmin’	0.156	

Correction method= # Reported T Limits: Tmin=0.493 Tmax=0.753
AbsCorr = MULTI-SCAN

Data completeness= 0.979 Theta(max)= 70.091

R(reflections)= 0.1858(5020) wR2(reflections)= 0.4271(5852)

S = 1.125 Npar= 318

The following ALERTS were generated. Each ALERT has the format
test-name_ALERT_alert-type_alert-level.
Click on the hyperlinks for more details of the test.

Alert level B

```
PLAT082_ALERT_2_B High R1 Value ..................................       0.19 Report
PLAT084_ALERT_3_B High wR2 Value (i.e. > 0.25) ...................       0.43 Report
PLAT213_ALERT_2_B Atom C13             has ADP max/min Ratio .....        4.8 prolat
PLAT341_ALERT_3_B Low Bond Precision on  C-C Bonds ...............     0.0206 Ang.
```

Alert level C

```
RINTA01_ALERT_3_C  The value of Rint is greater than 0.12
            Rint given   0.151
PLAT020_ALERT_3_C The Value of Rint is Greater Than 0.12 .........      0.151 Report
PLAT213_ALERT_2_C Atom S1              has ADP max/min Ratio .....        3.1 prolat
PLAT242_ALERT_2_C Low    'MainMol' Ueq as Compared to Neighbors of        C41 Check
PLAT244_ALERT_4_C Low    'Solvent' Ueq as Compared to Neighbors of        C39 Check
PLAT250_ALERT_2_C Large U3/U1 Ratio for Average U(i,j) Tensor ....        2.2 Note
PLAT906_ALERT_3_C Large K Value in the Analysis of Variance ......     17.448 Check
PLAT906_ALERT_3_C Large K Value in the Analysis of Variance ......      5.156 Check
PLAT906_ALERT_3_C Large K Value in the Analysis of Variance ......      2.986 Check
PLAT911_ALERT_3_C Missing FCF Refl Between Thmin & STh/L=    0.600         18 Report
```

Alert level G

```
FORMU01_ALERT_2_G  There is a discrepancy between the atom counts in the
            _chemical_formula_sum and the formula from the _atom_site* data.
            Atom count from _chemical_formula_sum:C60.3 H42.3 Cl12.9 Co1 N8 S2
            Atom count from the _atom_site data:  C60 H42 Cl12 Co1 N8 S2
CELLZ01_ALERT_1_G Difference between formula and atom_site contents detected.
CELLZ01_ALERT_1_G ALERT: Large difference may be due to a
            symmetry error - see SYMMG tests
           From the CIF: _cell_formula_units_Z    8
           From the CIF: _chemical_formula_sum  C60.30 H42.30 Cl12.90 Co N8 S2
           TEST: Compare cell contents of formula and atom_site data

           atom    Z*formula  cif sites diff
           C        482.40    480.00    2.40
           H        338.40    336.00    2.40
           Cl       103.20     96.00    7.20
           Co         8.00      8.00    0.00
           N         64.00     64.00    0.00
           S         16.00     16.00    0.00
PLAT004_ALERT_5_G Polymeric Structure Found with Maximum Dimension          2 Info
PLAT041_ALERT_1_G Calc. and Reported SumFormula    Strings  Differ     Please Check
PLAT051_ALERT_1_G Mu(calc) and Mu(CIF) Ratio Differs from 1.0 by .      4.38 %
PLAT068_ALERT_1_G Reported F000 Differs from Calcd (or Missing)...     Please Check
PLAT083_ALERT_2_G SHELXL Second Parameter in WGHT  Unusually Large     437.53 Why ?
PLAT171_ALERT_4_G The CIF-Embedded .res File Contains EADP Records          2 Report
PLAT300_ALERT_4_G Atom Site Occupancy of C16       Constrained at         0.5 Check
PLAT300_ALERT_4_G Atom Site Occupancy of C17       Constrained at         0.5 Check
PLAT300_ALERT_4_G Atom Site Occupancy of C18       Constrained at         0.5 Check
PLAT300_ALERT_4_G Atom Site Occupancy of C19       Constrained at         0.5 Check
PLAT300_ALERT_4_G Atom Site Occupancy of C20       Constrained at         0.5 Check
PLAT300_ALERT_4_G Atom Site Occupancy of C27       Constrained at         0.5 Check
PLAT300_ALERT_4_G Atom Site Occupancy of C28       Constrained at         0.5 Check
PLAT300_ALERT_4_G Atom Site Occupancy of C29       Constrained at         0.5 Check
PLAT300_ALERT_4_G Atom Site Occupancy of C30       Constrained at         0.5 Check
PLAT300_ALERT_4_G Atom Site Occupancy of C31       Constrained at         0.5 Check
```

```
PLAT300_ALERT_4_G Atom Site Occupancy of C32        Constrained at        0.6 Check
PLAT300_ALERT_4_G Atom Site Occupancy of C33        Constrained at        0.6 Check
PLAT300_ALERT_4_G Atom Site Occupancy of C34        Constrained at        0.6 Check
PLAT300_ALERT_4_G Atom Site Occupancy of C35        Constrained at        0.6 Check
PLAT300_ALERT_4_G Atom Site Occupancy of C36        Constrained at        0.6 Check
PLAT300_ALERT_4_G Atom Site Occupancy of C37        Constrained at        0.6 Check
PLAT300_ALERT_4_G Atom Site Occupancy of C21        Constrained at        0.4 Check
PLAT300_ALERT_4_G Atom Site Occupancy of C22        Constrained at        0.4 Check
PLAT300_ALERT_4_G Atom Site Occupancy of C23        Constrained at        0.4 Check
PLAT300_ALERT_4_G Atom Site Occupancy of C24        Constrained at        0.4 Check
PLAT300_ALERT_4_G Atom Site Occupancy of C25        Constrained at        0.4 Check
PLAT300_ALERT_4_G Atom Site Occupancy of C26        Constrained at        0.4 Check
PLAT300_ALERT_4_G Atom Site Occupancy of H16        Constrained at        0.5 Check
PLAT300_ALERT_4_G Atom Site Occupancy of H17        Constrained at        0.5 Check
PLAT300_ALERT_4_G Atom Site Occupancy of H19        Constrained at        0.5 Check
PLAT300_ALERT_4_G Atom Site Occupancy of H20        Constrained at        0.5 Check
PLAT300_ALERT_4_G Atom Site Occupancy of H27        Constrained at        0.5 Check
PLAT300_ALERT_4_G Atom Site Occupancy of H28        Constrained at        0.5 Check
PLAT300_ALERT_4_G Atom Site Occupancy of H30        Constrained at        0.5 Check
PLAT300_ALERT_4_G Atom Site Occupancy of H31        Constrained at        0.5 Check
PLAT300_ALERT_4_G Atom Site Occupancy of H33        Constrained at        0.6 Check
PLAT300_ALERT_4_G Atom Site Occupancy of H34        Constrained at        0.6 Check
PLAT300_ALERT_4_G Atom Site Occupancy of H35        Constrained at        0.6 Check
PLAT300_ALERT_4_G Atom Site Occupancy of H36        Constrained at        0.6 Check
PLAT300_ALERT_4_G Atom Site Occupancy of H37        Constrained at        0.6 Check
PLAT300_ALERT_4_G Atom Site Occupancy of H22        Constrained at        0.4 Check
PLAT300_ALERT_4_G Atom Site Occupancy of H23        Constrained at        0.4 Check
PLAT300_ALERT_4_G Atom Site Occupancy of H24        Constrained at        0.4 Check
PLAT300_ALERT_4_G Atom Site Occupancy of H25        Constrained at        0.4 Check
PLAT300_ALERT_4_G Atom Site Occupancy of H26        Constrained at        0.4 Check
PLAT301_ALERT_3_G Main Residue  Disorder .............(Resd  1  )        33% Note
PLAT335_ALERT_2_G Check Large C6 Ring C-C Range C15      -C31           0.22 Ang.
PLAT410_ALERT_2_G Short Intra H...H Contact  H8       ..H27      .       1.74 Ang.
                                                      x,y,z  =      1_555 Check
PLAT605_ALERT_4_G Largest Solvent Accessible VOID in the Structure        84 A**3
PLAT794_ALERT_5_G Tentative Bond Valency for Co1       (II)      .       1.94 Info
PLAT811_ALERT_5_G No ADDSYM Analysis: Too Many Excluded Atoms ....          ! Info
PLAT912_ALERT_4_G Missing # of FCF Reflections Above STh/L=  0.600        88 Note
PLAT955_ALERT_1_G Reported (CIF) and Actual (FCF) Lmax Differ by .         1 Units
PLAT978_ALERT_2_G Number C-C Bonds with Positive Residual Density.         5 Info
```

---

0 **ALERT level A** = Most likely a serious problem - resolve or explain
4 **ALERT level B** = A potentially serious problem, consider carefully
10 **ALERT level C** = Check. Ensure it is not caused by an omission or oversight
58 **ALERT level G** = General information/check it is not something unexpected

6 ALERT type 1 CIF construction/syntax error, inconsistent or missing data
10 ALERT type 2 Indicator that the structure model may be wrong or deficient
9 ALERT type 3 Indicator that the structure quality may be low
44 ALERT type 4 Improvement, methodology, query or suggestion
3 ALERT type 5 Informative message, check

It is advisable to attempt to resolve as many as possible of the alerts in all categories. Often the minor alerts point to easily fixed oversights, errors and omissions in your CIF or refinement strategy, so attention to these fine details can be worthwhile. In order to resolve some of the more serious problems it may be necessary to carry out additional measurements or structure refinements. However, the purpose of your study may justify the reported deviations and the more serious of these should normally be commented upon in the discussion or experimental section of a paper or in the "special_details" fields of the CIF. checkCIF was carefully designed to identify outliers and unusual parameters, but every test has its limitations and alerts that are not important in a particular case may appear. Conversely, the absence of alerts does not guarantee there are no aspects of the results needing attention. It is up to the individual to critically assess their own results and, if necessary, seek expert advice.

Publication of your CIF in IUCr journals

A basic structural check has been run on your CIF. These basic checks will be run on all CIFs submitted for publication in IUCr journals (*Acta Crystallographica*, *Journal of Applied Crystallography*, *Journal of Synchrotron Radiation*); however, if you intend to submit to *Acta Crystallographica Section C* or *E* or *IUCrData*, you should make sure that full publication checks are run on the final version of your CIF prior to submission.

Publication of your CIF in other journals

Please refer to the *Notes for Authors* of the relevant journal for any special instructions relating to CIF submission.

PLATON version of 05/12/2020; check.def file version of 05/12/2020

Datablock rod149c_0m - ellipsoid plot

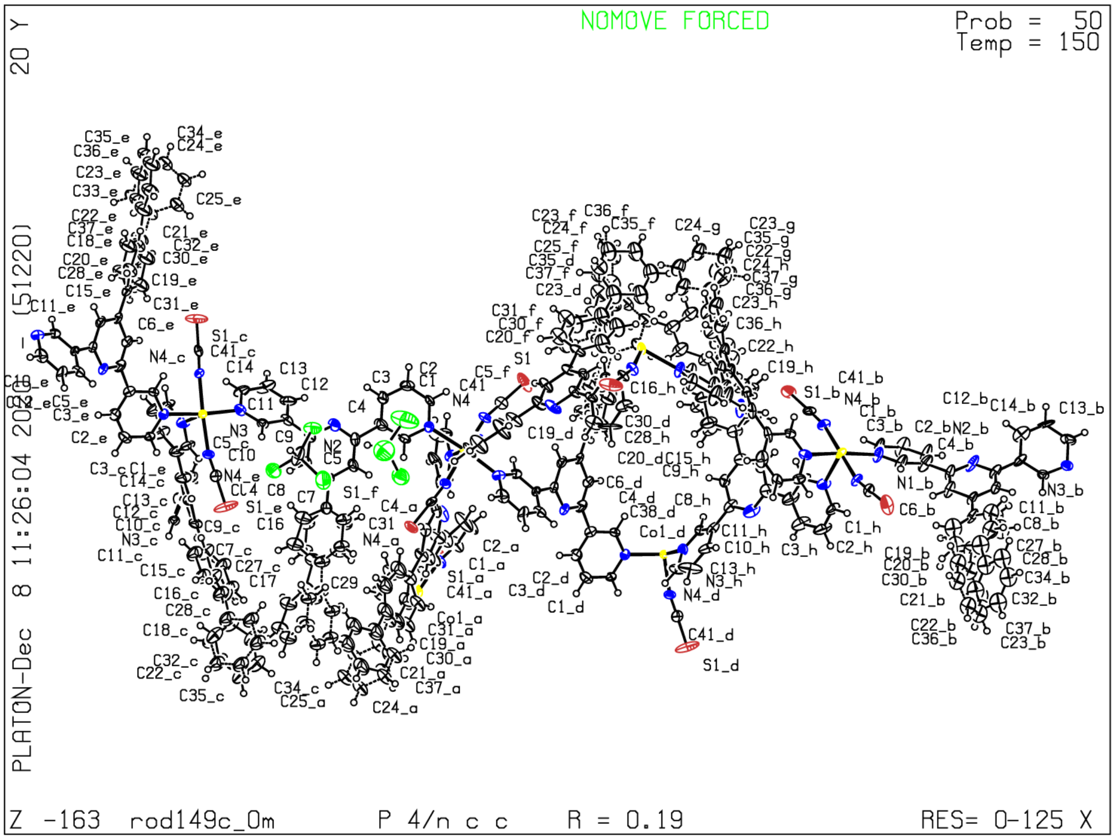

Supplement: Supplementary file 1 [file molecules-26-06337-s001.zip › supporting information-1434316-proof/ROD149C_0m_cifreport.pdf]
