# Supplementary material for: Adapting (4,4) Networks through Substituent Effects and Conformationally Flexible 3,2’:6’,3”-Terpyridines
Source: Molecules. 2021 Oct 20;26(21):6337. doi: 10.3390/molecules26216337 (PMC8587907; doi:10.3390/molecules26216337)
Supplement: Supplementary file 1 [file molecules-26-06337-s001.zip › supporting information-1434316-proof/Supporting Information 08Oct.pdf]

Supporting Information to accompany:

Adapting (4,4) Networks Through Substituent effects and Conformationally Flexible 3,2':6',3"-Terpyridines

Dalila Rocco, Alessandro Prescimone, Edwin C. Constable and Catherine E. Housecroft*

Department of Chemistry, University of Basel, Mattenstrasse 24a, BPR 1096, 4058 Basel, Switzerland

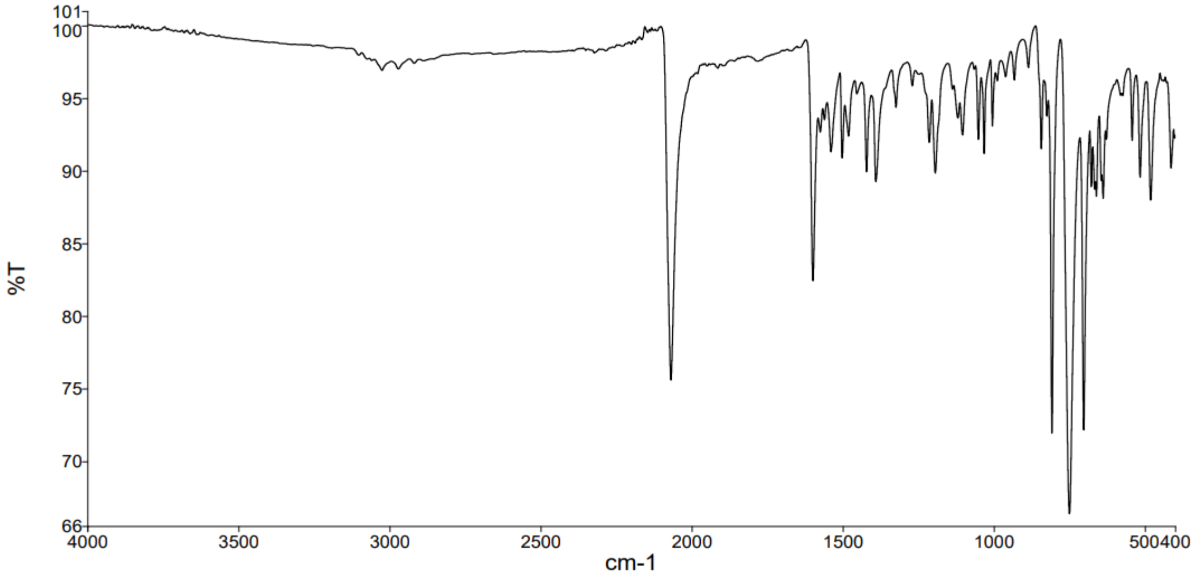

Fig. S1. Solid-state IR spectrum of $[Co(\mathbf{1})_2(NCS)_2]_n{\cdot}4.5n CHCl_3$

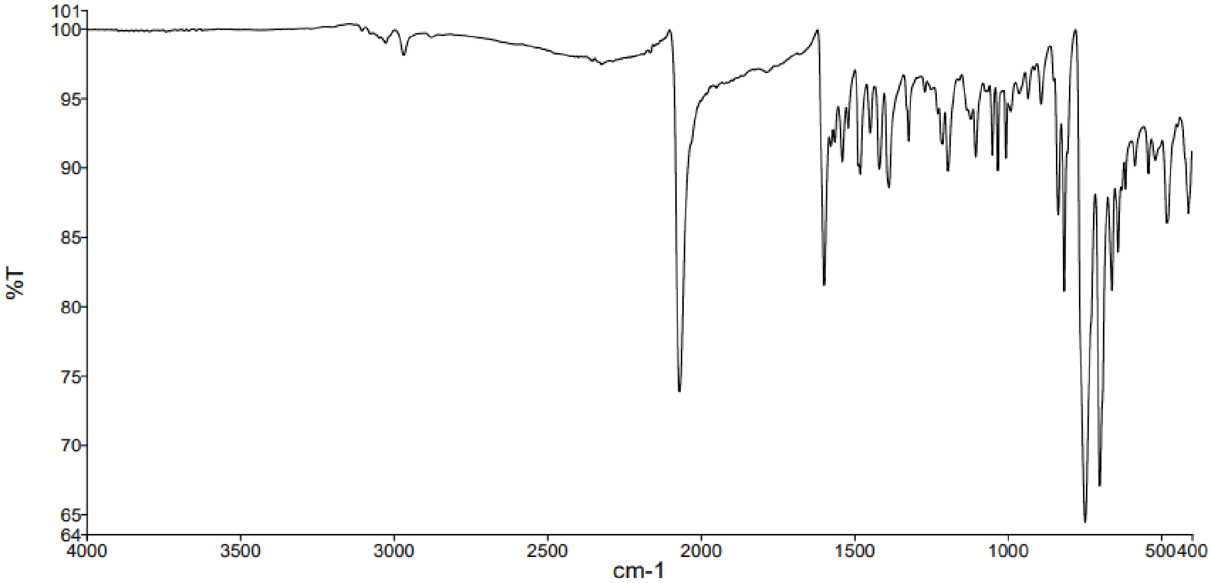

Fig. S2. Solid-state IR spectrum of $[Co(\mathbf{2})_2(NCS)_2]_n{\cdot}4.3n CHCl_3$

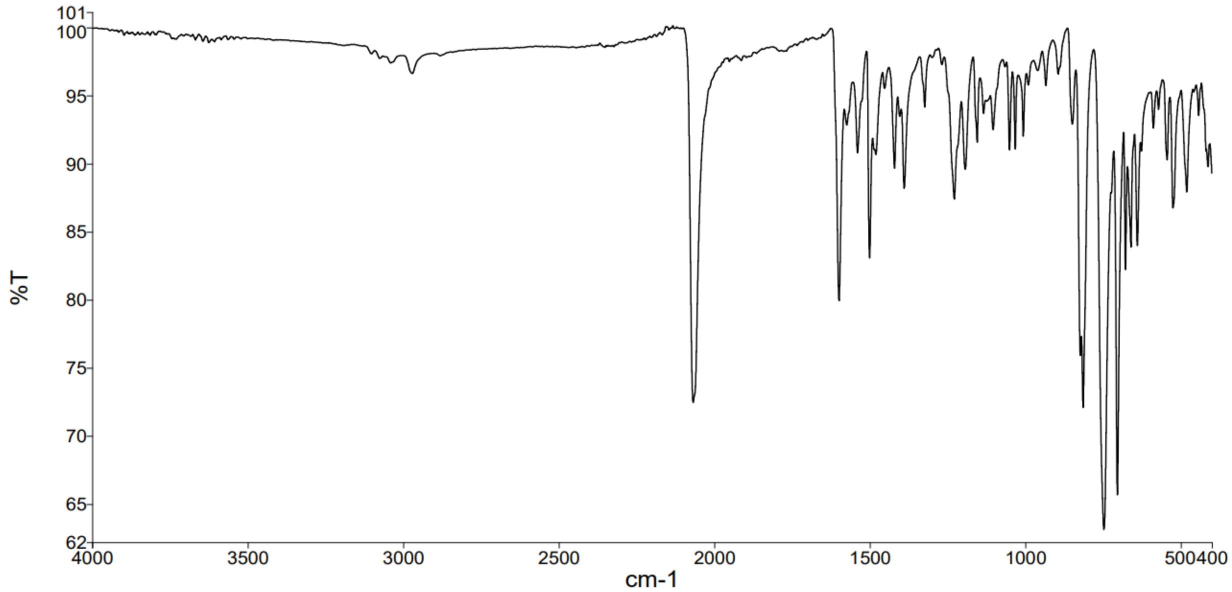

Fig. S3. Solid-state IR spectrum of $[Co(\mathbf{3})_2(NCS)_2]_n \cdot 4n CHCl_3$

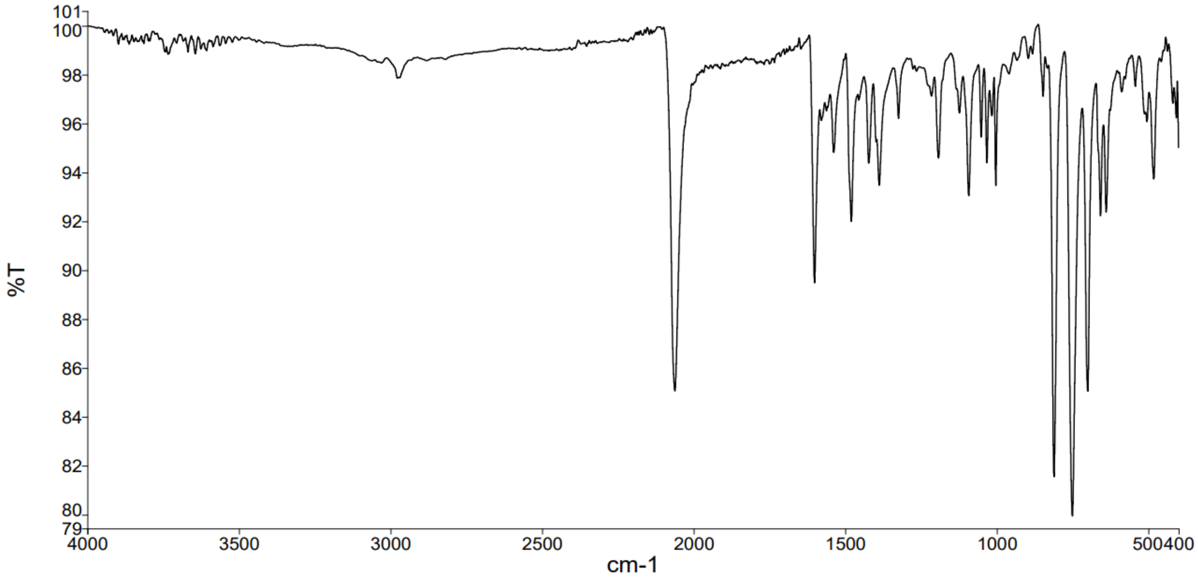

Fig. S4. Solid-state IR spectrum of $[Co(\mathbf{4})_2(NCS)_2]_n$

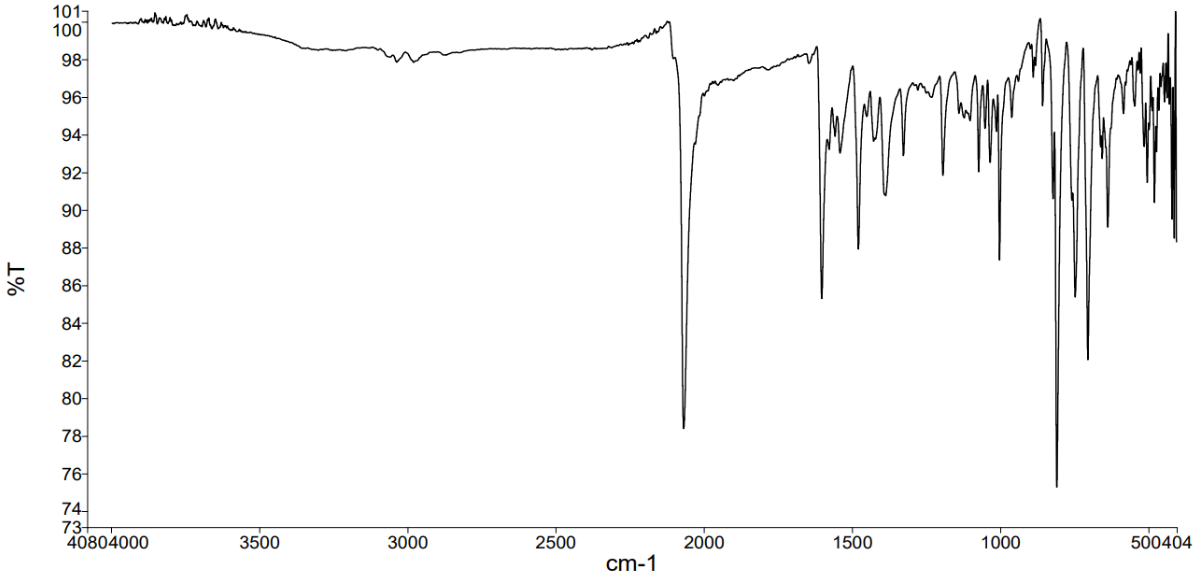

Fig. S5. Solid-state IR spectrum of $[Co(\mathbf{5})_2(NCS)_2]_n{\cdot}nCHCl_3$

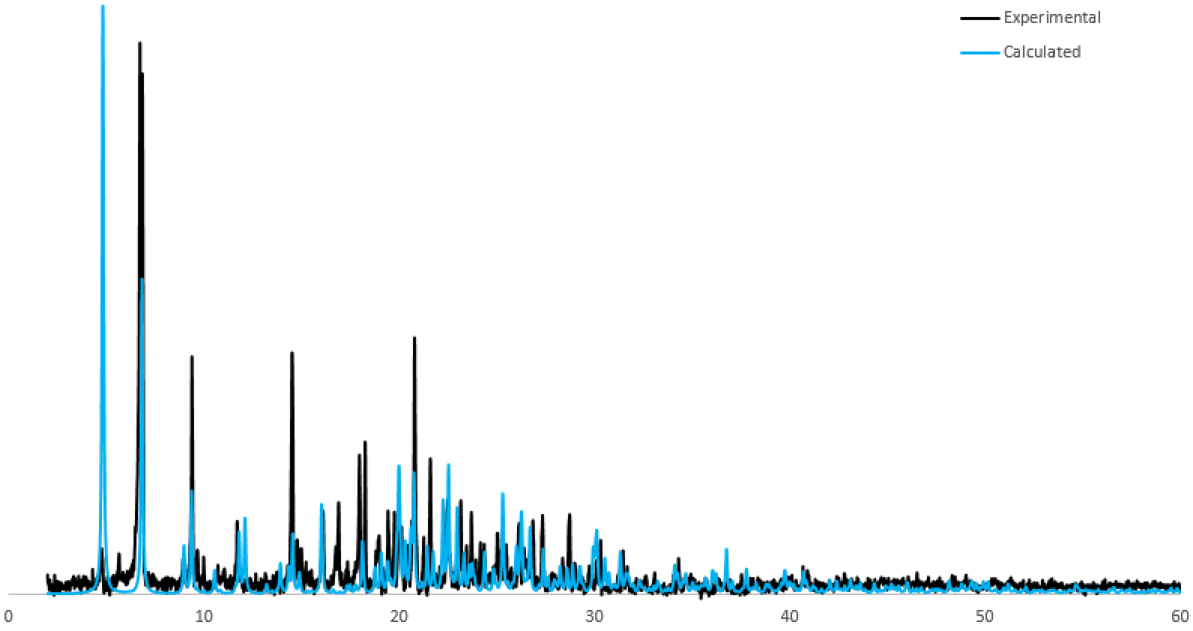

Fig. S6. Overlay of the experimental PXRD (298 K) for the bulk material and that predicted from the single crystal structure (150 K) of $[Co(\mathbf{2})_2(NCS)_2]_n{\cdot}4.3nCHCl_3$.

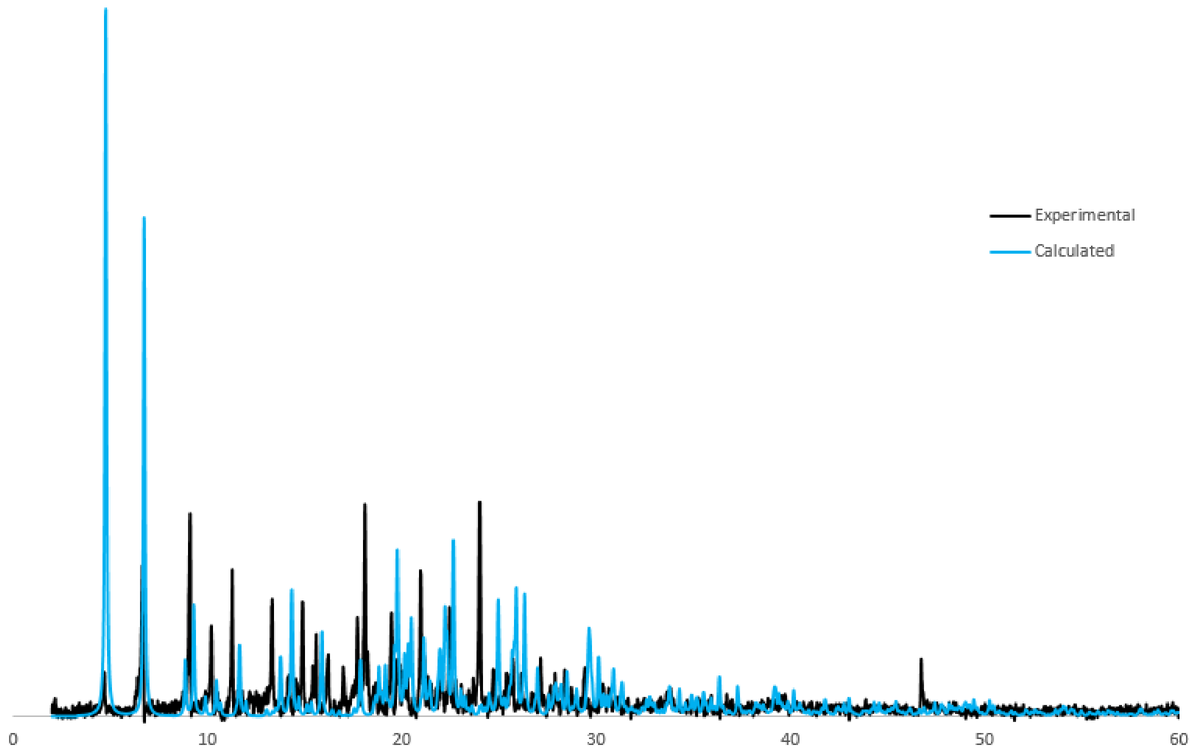

Fig. S7. Overlay of the experimental PXRD (298 K) for the bulk material and that predicted from the single crystal structure (150 K) of [Co(**1**)$_2$(NCS)$_2$]$_n$·4.5nCHCl$_3$.

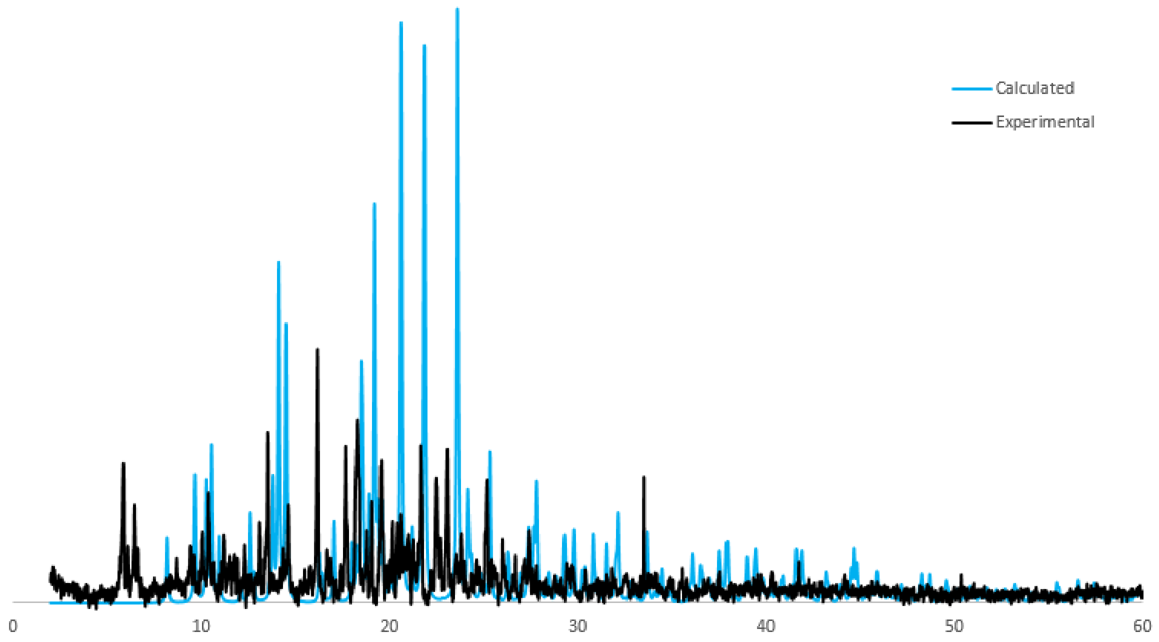

Fig. S8. Overlay of the experimental PXRD (298 K) for the bulk material and that predicted from the single crystal structure (150 K) of [Co(**4**)$_2$(NCS)$_2$]$_n$.

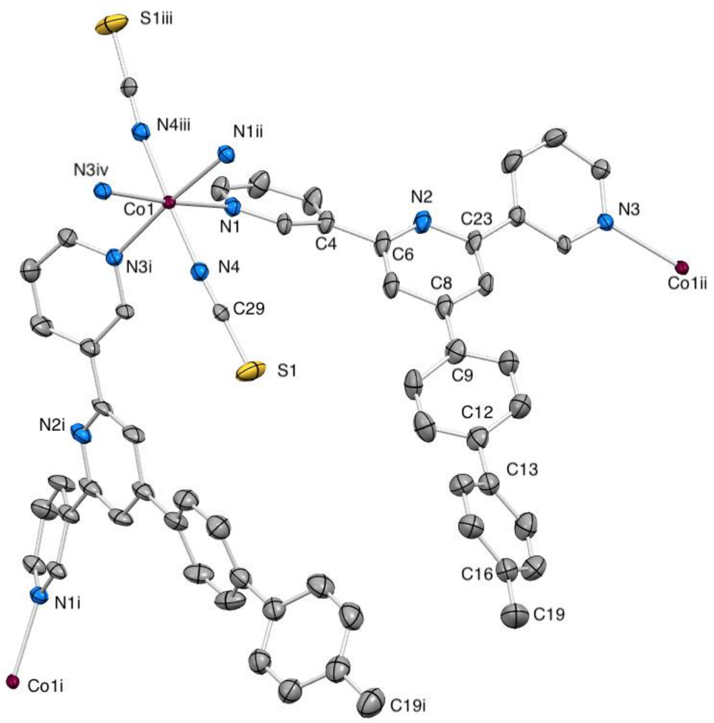

Fig. S9. Structure of the asymmetric unit in $[Co(\mathbf{1})_2(NCS)_2]_n{\cdot}4.5nCHCl_3$ with symmetry related atoms. Symmetry codes: i = ${}^{3}/_{2}$–*y*, *x*, *z*; ii = *y*, ${}^{3}/_{2}$–*x*, *z*; iii = 1–*y*, 1–*x*, ${}^{1}/_{2}$–*z*; iv = 1–*x*, –${}^{1}/_{2}$+*y*, ${}^{1}/_{2}$–*z*. H atoms and solvent molecules are omitted. Ellipsoids are plotted at 40% probability level.

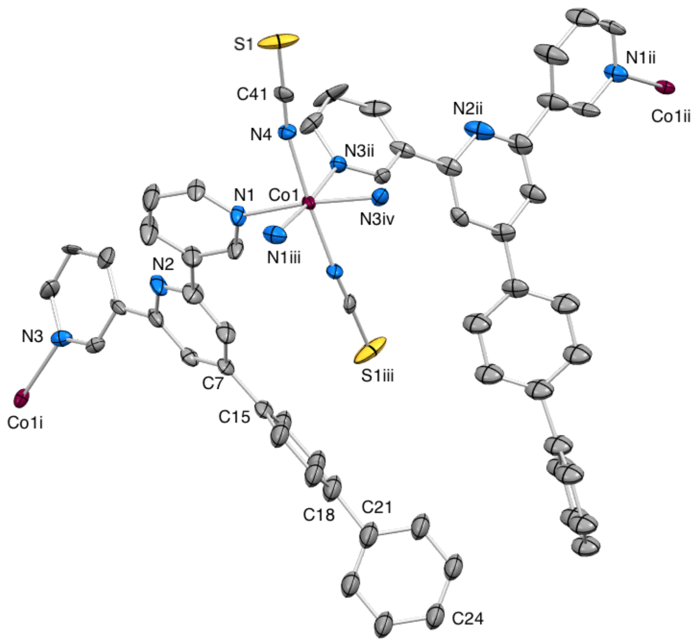

Fig. S10. Structure of the asymmetric unit in $[Co(\mathbf{2})_2(NCS)_2]_n{\cdot}4.3nCHCl_3$ with symmetry related atoms. The biphenyl unit is disordered (see text) and only one position is shown. Symmetry codes: i = *y*, ${}^{1}/_{2}$–*x*, *z*; ii = ${}^{1}/_{2}$–*y*, *x*, *z*; iii = –${}^{1}/_{2}$+*y*, ${}^{1}/_{2}$+*x*, ${}^{3}/_{2}$–*z*; iv = –${}^{1}/_{2}$+*x*, 1–*y*, ${}^{3}/_{2}$–*z*. H atoms and solvent molecules omitted. Ellipsoids are plotted at 40% probability level.

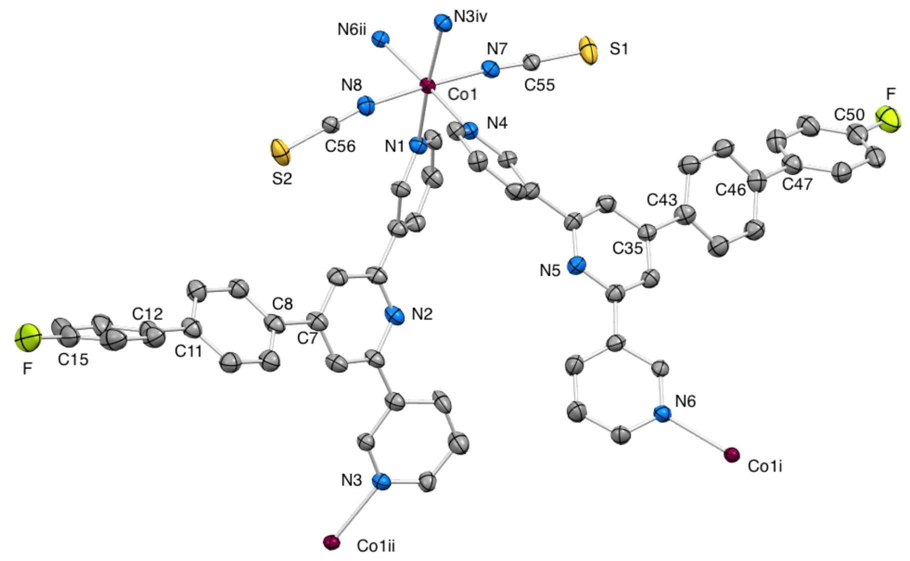

Fig. S11. Structure of the asymmetric unit in $[Co(\mathbf{3})_2(NCS)_2]_n{\cdot}4n CHCl_3$ with symmetry related atoms. H atoms and solvent molecules omitted. Ellipsoids are plotted at 40% probability level. Symmetry codes: i = $1-x$, $-^1/_2+y$, $^3/_2-z$; ii = $^1/_2+x$, $1-y$, $^3/_2-z$; iii = $1-x$, $^1/_2+y$, $^3/_2-z$; iv = $-^1/_2+x$, $1-y$, $^3/_2-z$.

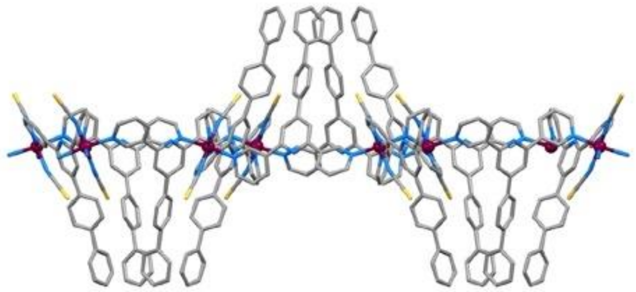

Fig. S12. Part of one 2D-sheet in $[Co(\mathbf{2})_2(NCS)_2]_n{\cdot}4.3n CHCl_3$ showing cones above and below the plane containing the Co atoms. H atoms and solvnet molecules are omitted.

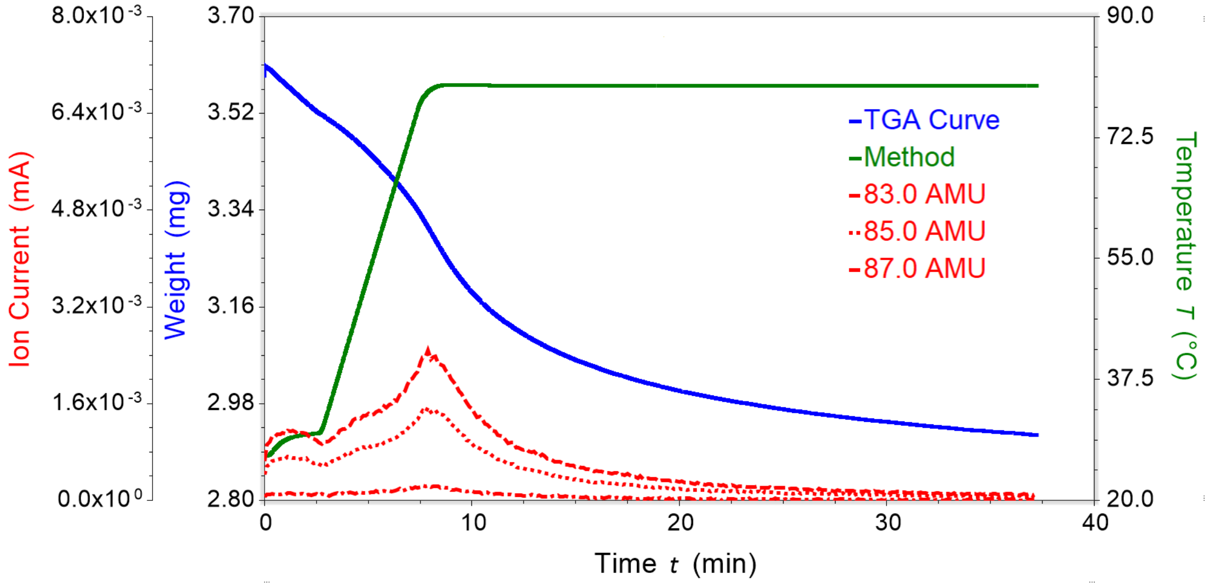

Fig. S13. TGA and mass spectrometric traces for the analysis of $[Co(\mathbf{3})_2(NCS)_2]_n{\cdot}4nCHCl_3$ (cycle 2: after 24 hour exposure to $CHCl_3$ vapor). Green: temperature vs. time; blue: weight of sample vs. time; red: mass detection for *m/z* 83.0 (most intense peak), 85.0 and 87.0. The initial mass of sample was 3.60 mg and a weight loss of 0.68 mg corresponds to ca. 19%.

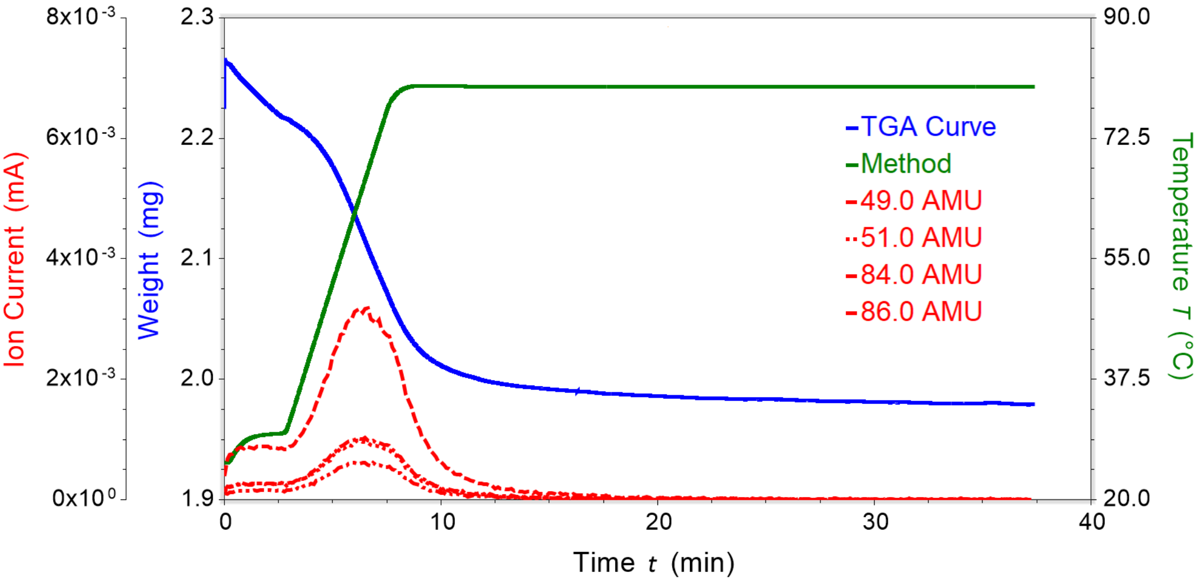

Fig. S14. TGA and mass spectrometric traces for the analysis of $[Co(\mathbf{3})_2(NCS)_2]_n{\cdot}2nCHCl_3{\cdot}2nCH_2Cl_2$ (cycle 4: after 24 hours exposure to CH_2Cl_2 vapor). Green: temperature vs. time; blue: weight of sample vs. time; red: mass detection for *m/z* 49.0, 51.0, 84.0, 86.0. The initial mass of sample was 2.26 mg and a weight loss of 0.28 mg corresponds to ca. 12%.

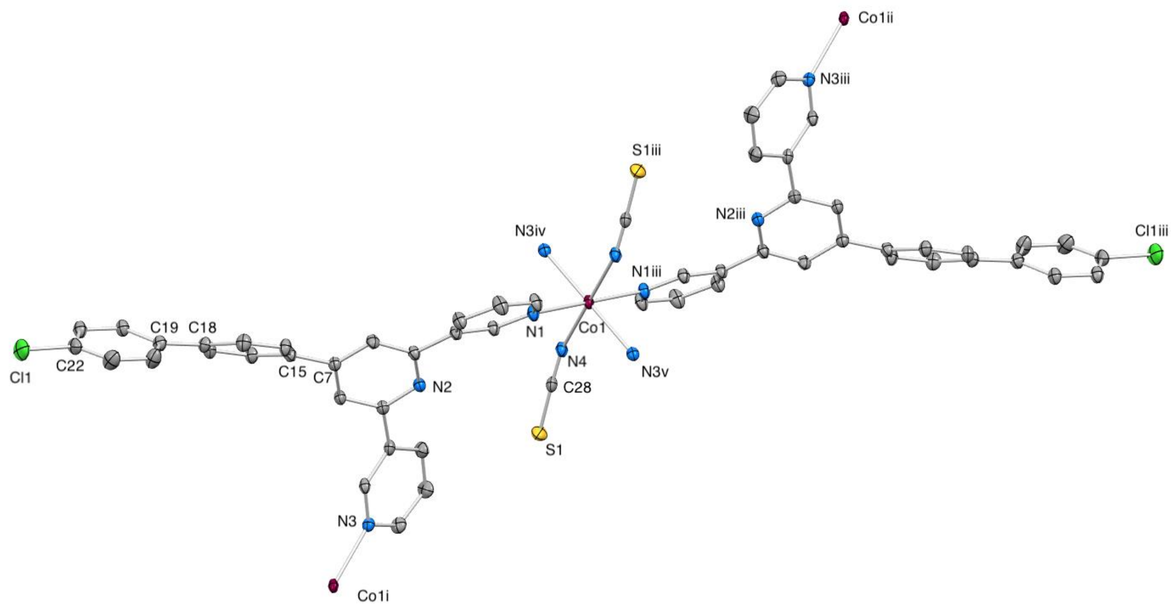

Fig. S15. Structure of the asymmetric unit in $[Co(\mathbf{4})_2(NCS)_2]_n$ with symmetry related atoms. H atoms and solvent molecules omitted. Ellipsoids are plotted at 40% probability level. Symmetry codes: i = $^{1}/_{2}+x$, $^{1}/_{2}+y$, $^{3}/_{2}-z$; ii = $-^{1}/_{2}-x$, $-^{1}/_{2}+y$, $^{1}/_{2}-z$; iii = $-x$, $-y$, $1-z$; iv = $-^{1}/_{2}+x$, $^{1}/_{2}-y$, $-^{1}/_{2}+z$; v = $^{1}/_{2}+x$, $-^{1}/_{2}+y$, $^{3}/_{2}-z$.

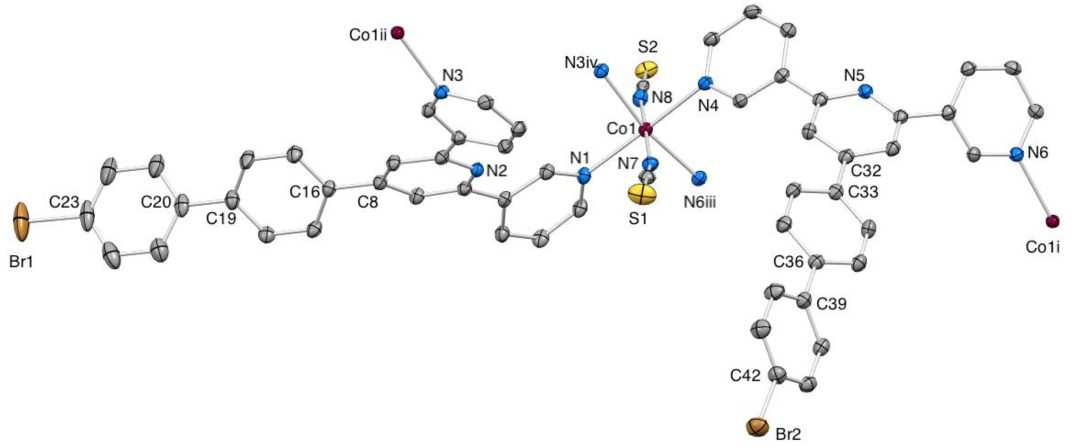

Fig. S16. Structure of the asymmetric unit in $[Co(\mathbf{5})_2(NCS)_2]_n{\cdot}nCHCl_3$ with symmetry related atoms. H atoms and solvent molecules omitted. Ellipsoids are plotted at 40% probability level. Symmetry codes: i = $^{5}/_{2}-x$, $^{1}/_{2}+y$, $^{1}/_{2}-z$; ii = $^{3}/_{2}-x$, $-^{1}/_{2}+y$, $^{1}/_{2}-z$; iii = $^{5}/_{2}-x$, $-^{1}/_{2}+y$, $^{1}/_{2}-z$; iv = $^{3}/_{2}-x$, $^{1}/_{2}+y$, $^{1}/_{2}-z$.
